# Living well with advanced cancer: a scoping review of non-pharmacological supportive care interventions

**DOI:** 10.1007/s11764-024-01714-z

**Published:** 2024-12-16

**Authors:** Brinda Kumar, Moe Thet Htaa, Kim Kerin-Ayres, Andrea L. Smith, Judith Lacey, Sarah Bishop Browne, Suzanne Grant

**Affiliations:** 1 School of Medicine, Western Sydney University, Campbelltown, NSW Australia; 2 Supportive Care and Integrative Oncology, Chris O’Brien Lifehouse, Sydney, NSW Australia; 3 The Daffodil Centre, University of Sydney, a joint venture with Cancer Council NSW, Sydney, NSW Australia; 4 NICM Health Research Institute, Western Sydney University, Westmead, NSW Australia; 5 Translational Health Research Institute (THRI), Western Sydney University, Campbelltown, NSW Australia

**Keywords:** Supportive care, Advanced cancer, Metastatic cancer, Cancer survivorship

## Abstract

**Purpose:**

The increasing number of people living longer with advanced cancer presents unique physical, psychosocial, financial, legal, practical and complex care needs. Supportive care interventions aim to address these needs by improving symptom management, promoting wellbeing, enhancing quality of life and potentially improving prognosis. To integrate supportive care interventions into clinical practice, a comprehensive review of existing studies is needed. This scoping review maps the evidence on non-pharmacological supportive care interventions for people with advanced cancer and identifies gaps to inform future research.

**Methods:**

We systematically searched four electronic databases—CINAHL, Medline, Cochrane and PsycINFO—for peer-reviewed original research on non-pharmacological supportive care interventions for adults with advanced cancer, published from January 1, 2013, to July 1, 2024.

**Results:**

Out of 3716 studies, 84 publications met the inclusion criteria. These studies were categorised into key supportive care domains: physical activity, psychosocial support, patient care and autonomy, multimodal approaches and others. Most publications focused on interventions addressing physical and psychosocial needs, showing benefits such as reduced fatigue, pain and improved mood. However, significant gaps were found in research on interventions addressing practical needs essential to autonomy, including health system and information needs, patient care and support and financial needs.

**Conclusion:**

Mapping the studies to the needs of the advanced cancer population showed that domains with greatest unmet needs have the fewest interventions available. Our scoping review suggests that non-pharmacological supportive care interventions can improve the wellbeing and quality of life of people living with advanced cancer. However, addressing methodological limitations requires further large-scale, multi-centre studies focusing on the identified gaps to inform the implementation of suitable supportive care programs.

**Implications for Cancer Survivors:**

Non-pharmacological interventions can boost wellbeing and quality of life for advanced cancer survivors, but addressing gaps in practical and systemic support is crucial.

**Supplementary information:**

The online version contains supplementary material available at 10.1007/s11764-024-01714-z.

## Introduction

For the most commonly diagnosed cancers—breast, prostate, lung, colorectal, bladder and melanoma—between 30 and 72% of patients will present with, or progress to, metastatic disease [1]. Advances in cancer therapies have contributed to longer survival in individuals with metastatic disease, and as access to treatment options like immunotherapy continues to expand, the population of people living with advanced cancer is expected to grow [2, 3]. “Advanced cancer” in this context typically refers to individuals with metastatic disease or those with “treatable but not curable” cancer, where the disease is unlikely to be eradicated but managed through therapies that slow progression, extend survival and aim to control symptoms and side effects associated with the cancer and its treatments [4]. The advanced cancer population experiences a high symptom burden, with common physical symptoms such as pain, fatigue, dyspnoea and gastrointestinal disturbances, alongside psychological challenges like anxiety and depression. These symptoms often follow unpredictable trajectories, significantly affecting daily functioning, quality of life and adherence to treatments [5–7]. Economically, advanced cancer imposes substantial direct and indirect costs: patients frequently face out-of-pocket expenses for supportive care, while the cumulative social and healthcare system costs [8, 9]. Although this population demonstrates a strong motivation to engage in supportive care, there is a paucity of evidence regarding safe and effective care provision and further research is needed [10, 11]. A comprehensive review of non-pharmacological supportive care interventions is needed to identify approaches that enhance quality of life, symptom management and overall wellbeing, with the goal of facilitating translation into clinical practice.

Historically, healthcare models for individuals with advanced cancer have been misaligned with the needs of this patient population, focusing predominantly on end-of-life care or on those with a prognosis of no more than 6 to 12 months [12]. Typical palliative care and survivorship care approaches may not address the unique combination of psychological distress, financial burden and the combination of acute and chronic symptoms that is experienced over a longer period of time [10, 13]. However, there is an increasing recognition of the importance of interventions that provide on-going, personalised care tailored to the unique and evolving needs of this group [11, 13, 14].

Non-pharmacological supportive care interventions can address symptoms that fluctuate due to varying treatments and disease progression to meet unique needs of this patient group. Definitions of what therapies are included under the umbrella of supportive care in people with cancer vary but there is some consensus, that supportive care aims to address physical, emotional, social and spiritual needs, the management of treatment side effects and practical concerns such as finance or information on managing future symptoms [10, 15–17]. Supportive care is delivered by multiple disciplines including allied health, social workers, psychologists, exercise physiologists and physiotherapists and increasingly integrates evidence-based mind–body practices and lifestyle modifications [16, 18].

Despite the benefits of supportive care, it is unclear what supportive care interventions are being investigated specifically for individuals living with advanced cancer. This scoping review aimed to map the characteristics of available evidence regarding non-pharmacological supportive care interventions in people living with advanced cancer and identify gaps to inform future research.

The following research questions were formulated:

1. What does the published evidence tell us about non-pharmacological supportive care interventions for people living with advanced cancer?
2. What are the gaps in the literature?

## Methods

This scoping review followed the Arksey and O’Malley framework [19] which has been used in similar studies. A protocol was developed prior and registered with the Joanna Briggs Institute (JBI) on 2023–08-21. We have reported the review according to the PRISMA Extension for Scoping Reviews (PRISMA-ScR) (Supplemental Materials 1) [20].

### Search strategy

A literature search was conducted across four databases, CINAHL, Medline, Cochrane and PsycINFO, to identify relevant papers published between January 1, 2013, and July 1, 2024. This study period was selected to coincide with newer targeted therapies that have become available allowing people with advanced cancer to live longer [21]. A comprehensive search strategy was developed in consultation with an experienced academic librarian and by adapting the search strategy of a scoping review of unmet needs describing a similar population [22]. The final search strategy for Medline is provided in Supplemental Materials 2.

### Inclusion criteria

#### Population

Studies that include adults (aged eighteen years or older) with advanced cancer of any cancer type and receiving active supportive cancer care. “Advanced cancer” refers to those diagnosed and living with metastatic disease or with “treatable but not curable cancer” which refers to the expectation that the cancer is highly unlikely to be eradicated with a high chance this cancer will lead to death [23].

Studies with a focus on patients receiving end of life care were not included. Papers that included a mixed sample of patients (i.e., patients at any cancer stage) were excluded except for papers separating results for advanced cancer patients, which permitted subgroup analysis.

#### Intervention

Any non-pharmacological supportive care intervention aimed at addressing physical, emotional, spiritual, social, quality of life, wellbeing, financial and informational needs of people with advanced cancer were included. No delivery or geographical limitations were applied, and interventions could include technology-based interventions (e.g., apps), in-person interventions, or a combination. We excluded support groups as this model of care was recently reviewed elsewhere [24]. Pharmacological and palliative care service interventions were excluded.

#### Comparison

All comparisons were included, including comparisons to no intervention or another intervention form.

#### Outcomes

All outcome measures were included, such as quality of life scales, pain measures and self-efficacy measures.

### Study design

We included original research articles that were quantitative or mixed-method studies to explore the full extent of original research. Non-original studies, such as editorials, abstracts without full papers and opinion pieces were excluded. Qualitative studies were also excluded due to the extensive nature of the quantitative literature and to maintain the clarity of the analysis. Reviews, including systematic reviews and meta-analyses, were excluded as they are not reporting primary data. However, their reference lists were cross-checked, in addition to snowballing, to identify other potential studies for inclusion.

### Data management and study selection

Articles meeting the inclusion criteria were downloaded into Endnote 20 citation management software and exported into Covidence. Titles and abstracts of all articles were independently screened against inclusion criteria by a pair of authors (SG, BK, KKA, MTH). This process was repeated for the full-text review, and the two authors required a consensus at all stages. If disagreements occurred, a third author was consulted to ensure consistency. At the beginning of each screening level, a calibration exercise for 20% of the sample was used to ensure a minimum interrater agreement of 80% [25].

### Data extraction

A data extraction template was jointly developed by two authors (BK, SG) using Covidence. Before extracting data, these authors piloted the template on five studies. Following piloting, reviewers discussed if modifications were required to ensure the template captured all relevant data.

Data extraction relevant to our aims include (1) article details including author and publication year; (2) participant criteria including cancer type, stage (e.g. advanced), age, gender, number of participants, location (where the patient receiving care was physically located when receiving the intervention, e.g., outpatient clinic, home); (3) study information including study design, aim and duration; (4) nature of intervention including intervention type (e.g., physical activity), intervention group and control group; (5) the supportive care need(s) addressed by the intervention (6) outcomes (primary and secondary study outcomes, study limitations and adverse events). Data were grouped by the type of supportive care intervention, with results presented in a table (Supplemental Material 3).

No quality or bias assessments were conducted. We mapped our intervention types according to Edney, Roseleur [22] which recognised three broad groups of needs for the advanced cancer population, namely, physical, psychosocial and practical needs, which includes financial and informational needs such as patient autonomy. This method of classification has allowed us to draw comparisons between the types of interventions available and the areas of unmet need identified in recently conducted studies [14].

## Results

**Fig. 1:**
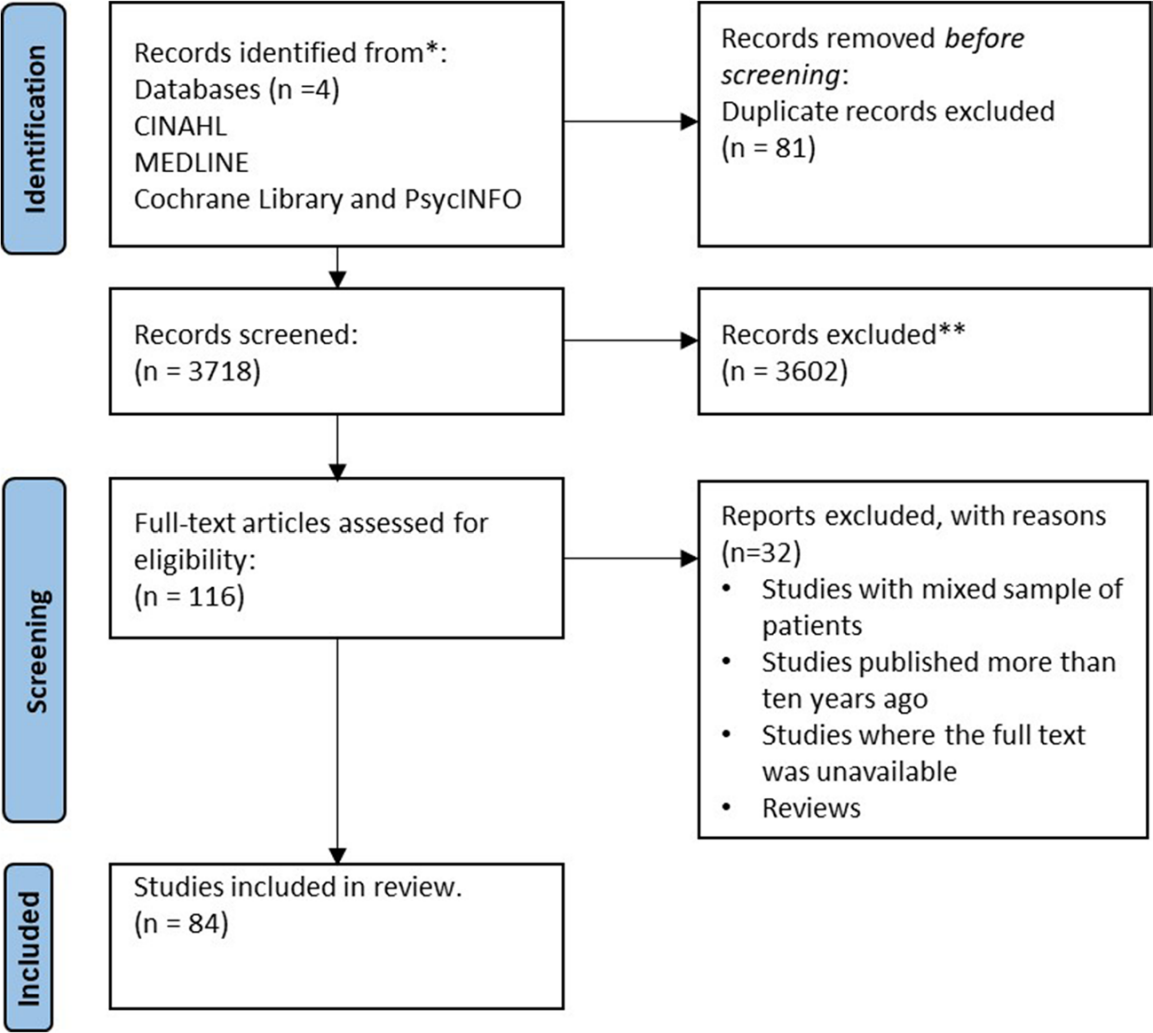
PRISMA flow diagram including screening and reasons for exclusion during second round of title and abstract screening

**Fig. 2:**
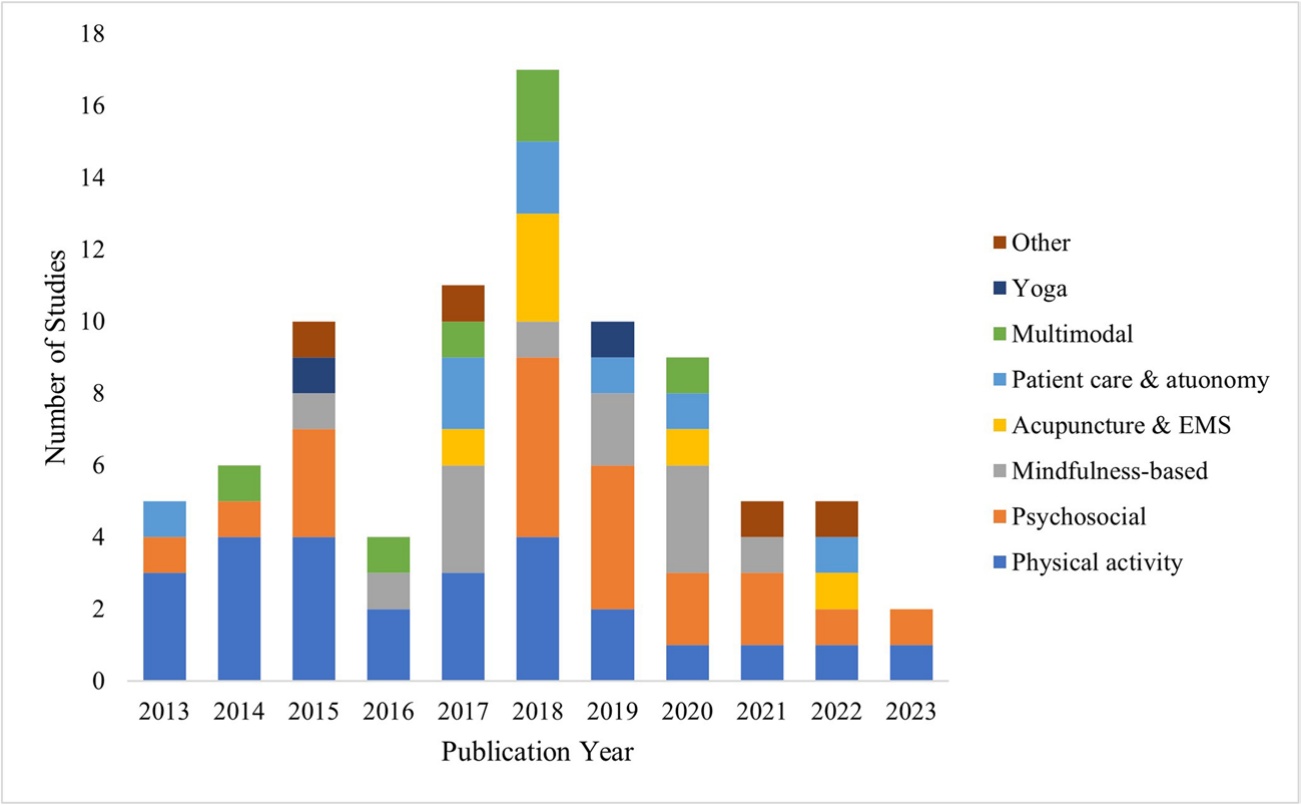
Distribution of peer-reviewed studies reporting non-pharmacological supportive care interventions of people with advanced cancer: 2013 to 2023

The search yielded 3716 studies. After title and abstract screening and removal of duplicates, 113 abstracts were retrieved for full-text evaluation. After examining full text, 84 publications were retained. Figure 1 shows the PRISMA flowchart of the study selection process. Sample and intervention characteristics are summarised in Fig. 2. Study characteristics are detailed in Supplemental Materials 3.

### Sample characteristics

#### Sample size

The sample size of the studies ranged from 6 to 349 participants, with a mean of 76 participants. The mean age of participants was 61.3 years. Eleven studies (13.1%, *n* = 11) had all female participants, 6 studies (7.1%, *n* = 6) had all male participants, and 67 studies (79.8%) included men and women. One study did not identify whether participants were male or female [26].

#### Cancer type

Forty-five studies focused on a single cancer type, including lung cancer (16.7%, *n* = 14), breast cancer (14.3%, *n* = 12), prostate cancer (7.1%, *n* = 6), melanoma (4.8%, *n* = 4), gastrointestinal tract cancer (3.6%, *n* = 3), colorectal cancer (3.6%, *n* = 3), ovarian cancer (2.4%, *n* = 2) and nasopharyngeal cancer (1.2%, *n* = 1). A population of mixed cancer types was the most common overall (46.4%, *n* = 39).

### Study design

#### Study type

Study designs included randomised controlled trials (RCTs) (73.8%, *n* = 62), single-arm interventional studies (15.5%, *n* = 13), mixed methods studies (3.6%, *n* = 3), two-arm interventional studies (2.4%, *n* = 2), retrospective clinical control trials (CCT) (2.4%, *n* = 2) and pilot studies (2.4%, *n* = 2).

#### Study settings

Intervention settings were varied and, in some instances, included multiple locations (6%, *n* = 5). Single-intervention locations include hospitals (45.2%, *n* = 38), outpatient clinics (16.7%, *n* = 14), participant homes (15.5%, *n* = 13), academic settings (9.5%, *n* = 8), gym or exercise area (4.8%, *n* = 4) and hospice (2.4%, *n* = 2).

#### Study length

The average intervention duration was 10 weeks, with a range of 3 days to 56 weeks.

#### Study mode of delivery

Studies were conducted in person (83.3%, *n* = 70), online (14.3%, *n* = 12), or a combination of both (2.4%, *n* = 2). Interventions were conducted individually (89.3%, *n* = 75), through group interventions (95.2%, *n* = 8), or a mix of both (11.9%, *n* = 1).

### Intervention types and outcomes

Most studies investigated a single intervention (92.9%, *n* = 78) and six studies (7.1%, *n* = 6) investigated multi-modal interventions. Only 34 studies (40.5%, *n* = 34) reported adverse events. Of these, most recorded no adverse events (33.3%, *n* = 28) or fatigue or distress directly related to the intervention (7.1%, *n* = 6). Thirty-two studies (38.1%, *n* = 32) showed statistically significant improvements in investigated interventions addressing psychosocial and physical supportive care needs through improvements in symptom burden and quality of life. Only one study reported adverse events as a primary outcome [27].

### Physical activity–based interventions

Twenty-six studies (31%, *n* = 26) focused on physical activity–based interventions alone, with twenty-one RCTs, three single-armed interventional studies and two two-armed interventional studies [28, 29]. Interventions included endurance [30, 31], strength [32] and resistance training [29, 33] with thirteen studies using more than one type of physical training (15.5%, *n* = 13) [26, 28–30, 32, 34–41]. Two studies explored isometric training of vertebral muscles [42, 43]. Other modalities included aerobic exercise [44, 45], very low interval training [46], walking interventions [47] and multifaceted programs prompting patients to exercise via text messages [48].

Two studies (2.4%, *n* = 2) investigated the feasibility of yoga interventions to improve quality of life. A couples-based Vivekananda Yoga (VKC) was tested in a single-armed feasibility trial on patients and their caregivers, assessing pre- and post-intervention levels of fatigue, sleep disturbances, psychological distress and relational closeness [49]. The other was an RCT focusing on mindful yoga techniques [50].

Of the twenty-six studies investigating physical activity–based interventions, certain studies demonstrated improvements in activity levels [29, 41, 48, 51], strength [30, 38], mobility [32], endurance [30] and reductions in pain and fatigue [38, 39]. High adherence rates were observed in programs suggesting feasibility and acceptability [34, 39]. Some interventions [35, 50] showed no significant change in fatigue, suggesting limited efficacy in addressing this symptom (*p* > 0.05). Multidimensional interventions [36, 41] provided further insight into exercise capacity improvements, highlighting the potential benefits of these interventions (*p* < 0.05). Further studies [26, 43] highlight the importance of high completion rates in attaining positive outcomes. Mixed findings and negative outcomes were also evident including challenges in recruitment and participation [46, 52].

Primary outcomes for the twenty-six studies investigating physical activity–based interventions, included activity levels [29, 41, 48, 51], strength [30, 38], mobility [32], endurance [30], lung capacity [36, 40], quality of life [26, 28, 42, 44] as well as reductions in pain and fatigue [26, 38]. Of the eight studies reporting feasibility, feasibility primary outcome criteria were completion rates [39, 43], adherence and attendance [34, 45, 46], adverse events [33] and satisfaction [47, 53].

### Psychosocial-based interventions

Psychosocial-based interventions include targeted interventions that address fear of cancer recurrence, mindfulness and distress through approaches such as cognitive behaviour therapy (CBT). Twenty studies (29.8%, *n* = 25) investigated psychosocial-based interventions, including fourteen RCTs, four single-armed interventional studies, one single-arm mixed methods study and one retrospective study. These programs reported significant reductions in depression [54–56], spiritual well-being [57, 58], death-related distress [55, 59, 60], sleep [61] and physical symptom distress [62–64]. Feasibility studies reported on satisfaction [52], acceptability[65] and adherence [66].

CBT protocols were used in six studies for patients with insomnia, anxiety, depression and fatigue [52, 54, 61, 66–68] including CBT via a mobile app to improve anxiety, depression and quality of life [67]. Acceptance and Commitment Therapy (ACT) was investigated for functional well-being and fatigue in sessions conducted in-person or via telephone [61, 69]. One study focused on the combined effect of CBT and ACT on the impact on insomnia [61]. Other modalities applied Meaning-centred Psychotherapy (MCP) to address existential distress and spiritual well-being [58]. Several interventions aimed to reduce cancer-specific distress and improve quality of life including Cognitive Behavioural Stress Management (CBSM), ACT [61–63, 70] and Managing Cancer and Living Meaningfully (CALM) [56, 71, 72]. One study investigated logotherapy to help individuals acquire meaning in their lives [60] while Dignity Therapy (DT) [59, 73] was used to encourage self-reflection as a means to achieve spirituality and identify a purpose in life.

CBT-based interventions were associated with improved mood and quality of life, particularly for those with insomnia and fatigue (7.1%, *n* = 6). Significant improvements in fatigue were noted with at-home delivered CBT intervention [52]. A study that delivered CBT via a mobile app also found significant improvements in anxiety, depression and quality of life when compared to baseline [67]. A CBT feasibility study reported high adherence to lessons (70%) accompanied with high treatment satisfaction [66]. CBT sessions delivered concurrently with chemoradiotherapy also demonstrate lower depression and anxiety scores twenty-four months after completion [74]. CBT focusing on ACT reported significant improvements in sleep efficiency, sleep latency, worry and depression from baseline to 6 weeks [61]. CBT focusing on stress reduction and management reported fewer depressive symptoms, intrusive thoughts and improvements in emotional wellbeing [72].

Two feasibility studies (2.4%, *n* = 2) investigated interventions for fear of cancer recurrence. One acceptability and feasibility RCT (Fear-Less: A Stepped-Care Program) stratified participants according to need to individual sessions delivered by a clinical psychologist or to a self-management group, compared to usual care [65]. In the self-management group, 13/21 participants had a reduction of Fear of Cancer Recurrence (FCR) and 5/7 participants in the individual psychologist session group. The stepped-care intervention was found to be acceptable and feasible. The other study was a nurse-led single-armed mixed methods study exploring the feasibility of a fear-conquering videoconferencing sessions. The intervention met feasibility and acceptability criteria with a reduction score of 8 points and 19.1 points for fear of progression and cancer-related distress respectively [75].

Twelve studies (14.3%, *n* = 12) investigated mindfulness interventions; seven RCTs, two single-arm interventional studies and three mixed methods studies. Interventions included art therapy [76], mindfulness-based cognitive therapy (MBCT) [71], mindfulness-based stress reduction (MBSR) [77–80], Lessons in Linking Affect and Coping (LILAC) [81], Naikan and Morita therapy [59, 73] and meditation interventions [70, 82, 83]. Seven studies reported increased positive changes in acceptance, mood and anxiety (8.3%, *n* = 7) [70, 73, 77–79, 81, 82]. Mindfulness and acceptance were measured by the Mindful Coping Scale (MCS), the Acceptance and Action Questionnaire-II (AAQ-II) and the Meaning in Life Questionnaire (MLQ) [82]. Spiritual-wellbeing was assessed with the Functional Assessment of Chronic Illness Therapy-Spiritual well-being scale (FACIT-Sp) [49, 70, 76, 84], Patient Health Questionnaire (PHQ-9) [55] and the Spiritual Well-being Scale (SWB) [58, 83].

### Symptom management and autonomy

Eight studies (9.5%, *n* = 8) addressed the practical needs of patient autonomy in symptom management and education. Six of these studies were RCTs and two were mixed methods. Three studies involved accessing an app or a website via a personal device or laptop. One study [85] used an app to allow patients to report their symptoms daily, and another app educated women on improving quality of life (QoL) during chemotherapy through a game [86]. Additionally, websites such as “Together” [87] and “Loop” [88] support clinical collaboration. Programs such as the life review program [64] and Be Resilient to Breast Cancer (BRBC) [89] facilitate resilience and empowerment. Furthermore, a nurse practitioner–led trial focused on telemonitoring pain [90]. In addressing anxiety, depression and stress among individuals coping with cancer, a comprehensive guided self-help program known as Targeted Selection, Enhanced Care, Stepped Care (TES) was investigated in a cluster RCT [91].

The TES Program, patients reported that screening survivor experiences were easy to complete (98%), acceptable (100%) and were all likely to recommend the therapy to others. All participants who completed the intervention reported subjective improvements in fear of cancer recurrence levels, and all attributed these changes to therapy [91]. Web-based programs such as Loop, a tool for clinical collaboration, enabled patients to communicate asynchronously with members of their healthcare team [88]. Other feasible web-based tools include an intervention targeting cognitive behavioural stress management [72] and nurse-led tele-health delivered survivorship care [92].

### Multimodal interventions

A multimodal intervention combines multiple therapeutic approaches or techniques to address various aspects of a health condition simultaneously. Six studies (7.1%, *n* = 6) investigated multimodal interventions. Of the six studies, five were RCTs and one was a single-arm interventional study. Studies included aerobic exercises alongside dietary advice [84]; Wheel Balance Cancer Therapy (WBCT) consisting of dietary advice, acupuncture and daily meditation [93] and the effects of Whole-body Electro-myo-stimulation (WB-EMS) alongside controlled nutritional intake [94]. Another study explored the effects of balance, endurance and exercise training for advanced colorectal cancer patients [95]. CBT concomitantly with graded exercise therapy investigated effects of fatigue [68]. Finally, another program combined exercise with dietary advice to investigate effects for patients with metastatic melanoma [96].

Implementing aerobic and resistance exercise [84] alongside dietary advice resulted in significant improvements in Functional Assessment of Cancer Therapy-Prostate (FACT-P) scores post-supervised intervention (*p* < 0.05), though this was not sustained. The Wheel Balance Cancer Therapy (WBCT) regimen [97] reported notable overall survival rates of 63.6% and 24.2% at the ends of years 1 and 2, respectively. The intervention offering CBT (*p* = 0.012) alongside Graded Exercise Therapy (GET) [68] demonstrated significant fatigue reduction. WB-EMS training (utilising light dynamic physical exercises and electrical muscle stimulation) resulted in higher skeletal muscle mass (*p* = 0.022) [94]. The comprehensive exercise program, including endurance and balance training [95], led to significant improvements in the Trial Outcome Index (TOI) (*p* < 0.05). Finally, the combining multimodal therapy with immunotherapy reduced symptom burden [96].

### Other interventions

Two RCTs (2.4%, *n* = 2) examined the effects of Yarrow liver compress for those with cancer-related fatigue undertaking palliative radiotherapy [98, 99] while nutritional interventions employing individualised diets targeted weight loss due to cachexia [100]. A live music–based intervention explored self-rated relaxation in comparison to an MBSR [101]. We found no studies addressing other key areas identified as practical needs of the advanced cancer population, including financial and sexual needs [102].

Three studies (3.6%, *n* = 3) explored the effects of acupuncture on cancer-related fatigue, quality of life and pain relief. One study investigated the efficacy of self-applied acupressure in alleviating fatigue levels [103] while another explored moxibustion acupuncture’s potential in enhancing quality of life metrics [104]. Additionally, a third study examined the role of intradermal acupuncture in managing cancer-related pain [105].

Three studies (3.6%, *n* = 3) investigated the effects of transcutaneous electrical stimulation on pain and symptomatic relief of chemotherapy such as fatigue, nausea and vomiting [106–108]. Electrical stimulation interventions using Transcutaneous Electrical Nerve Stimulation (TENS) significantly reduced pain when compared to baseline (*p* < 0.01); however, it did not sustain lasting effects after 60 and 120 min [107]. Nerve Electrical Stimulation (NES) therapy had a significant reduction in nausea (*p* = 0.02), vomiting (*p* = 0.04) and appetite improvement (*p* = 0.02) [106] while Neuromuscular Electrical Stimulation (NEMS) treatment groups did not achieve better outcomes in cancer-related fatigue as measured by the MFI scale (*p* = 0.21) [108].

## Discussion

This scoping review maps the breadth of research on non-pharmacological supportive care interventions in the population of people living with advanced cancer. Our review identified diverse interventions focusing on a range of supportive care needs, particularly physical and psychosocial needs. There were clear gaps in study design, with only three studies using a mixed methods approach, few studies set in the community or evaluating group-based interventions. The increasing number of included studies published from 2013 until 2018 reflects this field’s emerging nature, while the decline following this period may reflect the impact of COVID-19 on clinical trials [109].

**Fig. 3:**
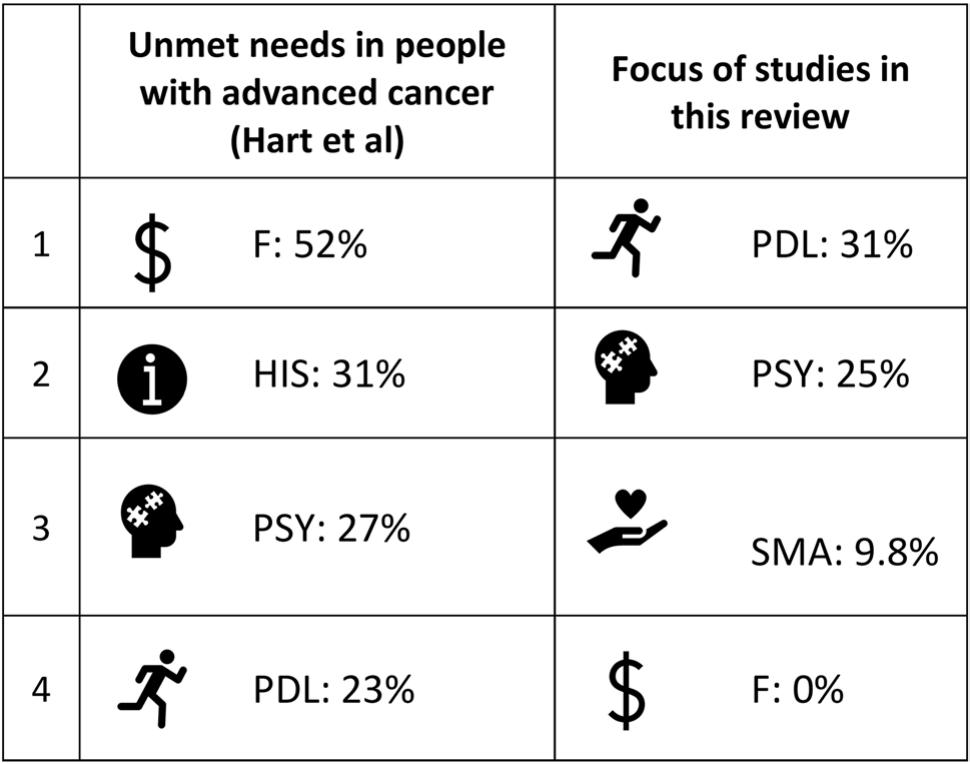
Areas of need versus focus of interventions. F, Financial; HIS, Health System and Information Needs; Psy, Psychological; PDL, Physical and Daily Living: SMA, Symptom management and autonomy

Most included studies addressed physical needs, such as fatigue, with clear benefits of structured exercise programs in improving physical activity levels and lean mass. However, there has been a rise in interventions addressing psychosocial needs, such as depression, over time. This follows an increased awareness of this population’s unique psychological challenges, including the long-term uncertainty of a life-limiting illness [110]. Feasible psychosocial approaches include coping skills and programs to reduce psychological distress and promote positive changes in acceptance. While physical and psychosocial needs remain well-addressed by interventions, an understudied domain is interventions addressing practical supportive care needs such as informational needs, financial needs and returning to work. Our findings align with existing studies that demonstrate these domains with greatest unmet need are the domains with fewest available interventions (see Fig. 1) & Fig. 3 [102]. Given its role in person-centred care, this warrants further research and the future development of programs addressing these unmet domains.

Few multimodal interventions were studied. In the context of advanced cancer, multimodal interventions might integrate physical therapies, psychological counselling and nutritional support to manage pain, reduce anxiety and improve overall quality of life. These interventions tailored to the individual’s unique needs may prove to be more effective than single-modality approaches, as they address the multidimensional nature of many health conditions.

Our review showed that most studies were conducted in hospital settings. While these hospital-based interventions are vital, the heterogeneity of the advanced cancer population and living with an incurable illness means that community interventions are vital. It follows that further research into optimising care pathways involving primary care providers in survivorship interventions is vital.

A growing area of service delivery is supportive care. Methods of incorporating technology in selected studies include web-based interventions and apps. Our findings are consistent with the advantages identified in existing literature including broader dissemination and cost efficiency while barriers include reduced medical record integration and healthcare provider participation [111]. As collaboration is vital to caring for patients with complex needs, addressing these limitations in future research is essential. The two identified app-based interventions [67, 85] demonstrated positive outcomes including high patient engagement, symptom control and continuity of care. While a self-management tool such as an app can increase patient empowerment [85], on-going research is required to optimise adherence.

While most studies utilised individual therapy, limited studies have used group therapy. One group intervention [60] effectively reduced existential concerns of loneliness. For this psychosocial need, group therapy uniquely positions patients to heal in a setting of shared empathy and hardship. Given these benefits, group therapy has suitability in other supportive care domains such as physical needs, particularly for motivation and should be an avenue for future research.

A significant gap identified by this scoping review is the methodological limitations of the evaluated supportive care interventions. Small sample sizes, short study duration and recruitment from only one site in most studies render most results preliminary and lacking statistical power. Finding appropriate control groups can be difficult, as patients may be receiving various concurrent therapies that impact outcomes. These methodological gaps and challenges restrict the ability to draw definitive conclusions about the efficacy of interventions, which hampers the development of comprehensive guidelines and impedes the implementation of supportive care for this population. To address this, large-scale, population-based research and novel research approaches are needed to build robust evidence and facilitate effective supportive care strategies for individuals living with advanced cancer [112].

### Strengths and limitations

This is the first scoping review to comprehensively synthesise evidence and identify gaps regarding non-pharmacological supportive care interventions for individuals living with advanced cancer. It excludes the typical pharmacological and palliative care service provision that has become current standard of care during acute treatment and in the patients with complex progressive disease. It was guided by a protocol based on expert scoping review methodology, utilised a search strategy developed with an academic librarian with inclusive and specific search terms and rigorous screening procedures to ensure all key studies were identified. This is a unique trait to this review, as existing studies neglected a full-text review due to the quantity of identified studies [22]. Another strength of this scoping review is its ability to capture a heterogeneous study population with different cancer types in different settings.

A barrier to the implementation of supportive care interventions in clinical practice is lack of access to synthesised evidence. Existing reviews of interventions for this population have targeted specific domains such as exercise and nutrition [113]. Such disaggregated reporting manifests as a limitation for clinicians in selecting interventions for their patients, as they cannot compare interventions addressing different care needs. Therefore, the breadth of this scoping review, accessibility of the results in a tabulated format and transparency to intervention outcomes and adverse events will improve quality of care by allowing clinicians to make informed clinical decisions and feel more confident in combining these interventions with standard care.

As an emerging field, there are inconsistencies surrounding the term advanced cancer which may have reduced identification of studies. Additionally, further relevant studies may have become available since conducting the search on July 1, 2024. While these are unlikely to significantly impact the conclusions drawn from this review, it remains a limitation as important interventions may not have been included. Despite using a comprehensive search strategy, all potential databases were not used, and therefore, all available literature may not be identified, Our review only included English terms and articles published in English, presenting a language bias and while in keeping with standard protocol for scoping reviews, the methodological quality of the studies was not assessed. Finally, excluding the paediatric population and an overall under-representation of the haematological cancers where there is no clear advanced stage limits the generalisability of these results.

### Future direction/recommendations

Our scoping review has highlighted the benefits of supportive care interventions for people with advanced cancer. Future research to assess the efficacy of supportive care interventions should be large multi-centre studies including community or primary care–based interventions and multi-modal interventions. These interventions should address the identified gaps including practical needs such as financial and informational needs and integrate methods to optimise and measure adherence to these interventions. Subsequent phases will involve optimising implementation by identifying barriers and facilitators to programs. Combining these findings with our review can support the development of intervention options.

The recently released MASCC-ASCO standards for supportive care for people with advanced or metastatic cancer provide seven standards and 45 practice recommendations to support optimisation of care experiences and health outcomes [114]. These standards highlight the importance not just of evidence-based and comprehensive supportive care, but of care that is person-centred, coordinated, integrated, accessible, equitable, sustainable and well-resourced. The standards reinforce the need not just to add to the evidence-base around effectiveness of supportive care interventions but to ensure that system-level factors such as patient navigation support, timely referrals to interprofessional supportive care services and models of care (e.g. specialist- vs nurse-led) meet the patient’s needs. Going forward, improvements in the experiences of people with advanced cancer and their health outcomes will require a coordinated response across multiple domains.

## Conclusion

The advanced cancer population is understudied and growing, experiencing a range of unmet care needs, which supportive care interventions can address. This scoping review has synthesised the published evidence and identified specific gaps where future research is required, with implications to improve outcomes for this population.

## Supplementary information

Below is the link to the electronic supplementary material.

Supplementary file1 (DOCX 114 KB)

Supplementary file2 (DOCX 54 KB)

Supplementary file3 (DOCX 20 KB)

## Data Availability

No datasets were generated or analysed during the current study.
