# Supplementary material for: Living well with advanced cancer: a scoping review of non-pharmacological supportive care interventions
Source: J Cancer Surviv. 2024 Dec 16;20(3):1119–30. doi: 10.1007/s11764-024-01714-z (PMC13144243; doi:10.1007/s11764-024-01714-z)
Supplement: Supplementary file 1 — Supplementary file1 (DOCX 114 KB) [file 11764_2024_1714_MOESM1_ESM.docx]

**Table 1. Study characteristics of physical activity interventions (n=26)**

| Author/Year | Intervention | Sample Characteristics | Cancer Type | Study Design | Study Setting | Study Duration | Mode of delivery | Study Outcomes | Key Findings |
| --- | --- | --- | --- | --- | --- | --- | --- | --- | --- |
| Bade et al, 2018 | Patient-centred activity regimen (PCAR).  I1: weekly phone call.  I2: twice daily text message.  Both groups wore FitBit Flex, I1 received phone calls weekly to remind them to exercise. I2 received twice-daily text messages, one educational session. | I1: *n*= 29  I2: *n*=15  Mean age= 66.4yrs  Female= 11 (30%) | Lung cancer Stage III or Stage IV | Non-RCT | University | I1: 4 wks  I2: 12 wks | In person  Individual | Primary – Daily step count  Secondary – feedback survey | I1, more active patients increased (ES = 0.08) less active patients decreased (−0.12) step counts. I2, more active patients decreased (ES = −0.09), less active patients increased (0.39) step counts. |
| Boileau et al, 2023 | Adapted physical activity (APA) questionnaire for all patients  I1: Consultation for home-based exercise  I2: Individual endurance, muscle training, stretching  I3: Supervised endurance, muscle training, stretching 45 mins x 2 weeks | I1: *n* = 37  I2: *n* = 36  I3: *n* = 15  Mean age= 61.3yrs  Female= 137 (50%) initial | Melanoma Stage III and Stage IV | Observational retrospective pilot study | University Hospital | 3 months | In person  Individual | Primary – Recruitment, adhesion  Secondary – FACT-M | Recruitment was 63.4% (n=45/72) of advanced melanoma patients and 61.5% (n=24/39) of stage IV melanoma. 2/12 did not participate > 70%. FACT-M was 129.47 ±22.80 (95% CI). |
| Cheung et al, 2021 | I1: Aerobic exercise (60 minutes twice/week + self-exercise 150 minutes/week. Aerobic: walking on a treadmill, cycling on a stationary bike, strengthening exercises  I2: Tai-chi: self-practice, and education.  I3: self-exercise 30 minutes x 3 times/week during the intervention and 5 times/week post intervention | I1: *n* = 10  I2: *n* = 9  I3: *n* = 11  I2: mean age= 61yrs  I2: mean age= 61.1yrs  I3: mean age= 58.36 Female= 14 (46%) | Lung cancer  Stage IIIB or Stage IV | 3-arm RCT (blinded) | Gym | 12 wks | In person  Group | Primary – Intervention completion  Secondary – PSQI, BFI | 80% of patients in I1 and 78% of patients I2 completed the intervention. |
| Cheville et al, 2013 | I: Rapid, Easy, Strength Training (REST) exercise program x 10 repetitions of each exercise twice a week for 4 wks and x 15 repetitions of each exercise twice a week for another 4 wks  C: Usual care  2 sets of five-exercise routines, one targeting the upper and the other the lower body at least twice a week. Participants wore Pedometers at least four days per week. | I: *n* = 33  C: *n* = 33 I: mean age= 63.8 yrs  C: mean age= 65.5 yrs Female= 31 (53%) | Colorectal or Lung cancer Stage IV | RCT (blinded) | Outpatient clinic | 8 wks | In person  Individual | Primary - AM-PAC Mobility and activities short form  Secondary - Ambulatory Post-Acute Care Daily Activities SF, FACTG | The intervention group had significant improvements in mobility (p=0.01), fatigue (p=0.02), and sleep quality (p=0.05). |
| Cormie et al, 2013 | I: Resistance exercise x 60 minutes twice weekly  C: Usual care  I: resistance exercise load progressed from 12–8 repetition maximum (RM) followed by Low-level aerobic exercise and stretching low-level aerobic exercise and stretching. | I: *n* = 10  C: *n* = 10  I: mean age= 73.1 yrs  C: *n* mean age= 71.2 yrs Female: 0 | Prostate cancer Advanced | RCT (1:1 Prospective pilot) | Exercise clinic | 12 wks | In person  Individual | Primary – Adverse events, VAS  Secondary – leg extension, 400-m walk, usual and fast pace 6-m walk | No adverse events or skeletal complications occurred during the exercise sessions. |
| Dhillon et al, 2018 | I: Individualised PA programme x 1 hour a week - aerobic, and home-based was encouraged. Exercise participants received a pedometer and diary.  C: Usual care | I: *n* = 56  C: *n* = 55  Median age= 64 yrs Female= 50 (49.5%) | Lung cancer Stage III/IV | RCT (1:1 open label study) | Outpatient | 2 months | In person  Individual | Primary – FACT-F  Secondary – EORTC-QLQ-C30, General Health Questionnaire-12 | No significant change in fatigue for both groups. |
| Galvao et al, 2018 | I: Modular multimodal exercise program (M3EP) x 3 times a week 60 minutes each session  C: Usual care  Resistance exercise targeting the upper and lower body muscle, aerobic exercise of walking, cycling, rowing; flexibility included static stretching. | I: *n* = 28  C: *n* = 29  I: mean age= 69.7 yrs  C: mean age= 70.4 yrs Female= 0 | Prostate cancer Advanced / Metastatic | RCT (Prospective) | University | 3 months | In person  Group | Primary – SF-36  Secondary – Leg extension, 6-m walk, 400-m walk, SOT | Intervention group had significant improvements in self-reported physical function (p= 0.028). |
| Henke et al, 2014 | I: Strength training x every other day, endurance training x 6 minutes 5 days a week, and breathing techniques.  C: Conventional physiotherapy  Endurance training was hallway and a stair walking exercise. The strength training included four different endurance strength exercises every other day and physiotherapeutic breathing. | I: *n*= 18  C: *n*= 11  Age= NA Female= NA | Lung cancer Stage III and IV | RCT (Prospective) | Hospital | 14 months | In person  Individual | Primary – Barthel Index  Secondary – EORTC QLA C-30/LC-13 | Significant differences between the groups were detected (p =.0003), for activities of daily living. |
| Jastrzębski et al, 2015 | I: Pulmonary rehabilitation nordic walking 45minutes x 5 days a week, aerobic and respiratory exercises 30 minutes x once a day, resistance training 30 minutes x once a day.  C: Respiratory muscles exercise and cycling | I: *n*= 12  C: *n*= 8 Median age= 65 yrs Female= 2 (10%) | Lung cancer Stage III and IV | RCT | Hospital | 8 wks | In person  Individual | Primary – FVC, FEV1  Secondary – MRC, BDI | Intervention had significant improvement in FEV1 (p = 0.016). There was no change for FVC. |
| Jensen et al, 2014 | I1: Resistance (RET) 45 minutes x twice a week, supervised training sessions  I2: Aerobic exercise training (AET) 45 minutes x twice a week.  Supervised sessions on a bicycle ergometer. | I1: RET*: n* = 11  I2: AET*: n* = 10  Mean age= 55 yrs Female= 11 (53%) | GIT cancer Advanced | Randomised- interventional study (pilot) | Outpatient clinic | 12 wks | In person  Individual | Primary - EORTC-QLQC30  Secondary – 1-RM | I2 had increased EORTC-QLQ-C30 scores (p=0.045) compared to I1. |
| Kuehr et al, 2014 | Combined endurance and resistance training program X 5 times a week for inpatient and 3 times a week for outpatient  Physical exercise with endurance, strength, 6-MWT, and handheld dynamometry. | *n* = 40 Mean age= 60 yrs Female= 16 (40%) | NSC Lung cancer Advanced | Single-arm interventional study (prospective pilot) | Hospital, home | 8 wks | In person  Individual | Primary – Adherence  Secondary – 6MWT, HHDM | 55% of the participants fulfilled the criterion of adherence. |
| Ligibel et al, 2016 | I: Moderate-intensity aerobic exercise program x 150 minutes a week  C: Wait-list routine care  The intervention was online and in- person with an exercise physiologist. | I: *n* = 47  C: *n* = 51  Mean age= 49 yrs Female= 100 | Breast cancer Advanced / Metastatic | RCT (1:1) | Home | 16 wks | In person  Individual | Primary – EORTC QLQC30, Bruce Ramp Treadmill Test  Secondary – EORTC QLQ-C30, FACIT | Non-significant improvement in physical functioning compared to control (p = 0.23) and for the Bruce Ramp Treadmill (p=0.35). |
| Litterini et al, 2013 | I1: Individualised resistance x 30-60 minutes twice a week  I2: Cardiovascular exercise x 30-60 minutes twice a week  Participants attended monitored resistance or cardiovascular exercise sessions. | I1: Cardiovascular: *n* = 32  I2: Resistance: *n* = 34  Mean age= 62 yrs Female= 36 (54%) | Mixed Advanced | 2-arm randomised interventional trial | Hospital | 10 wks | In person  Individual | Primary – SPPB  Secondary – VAS | SPPB increased significantly regardless of Group (P<.001). |
| Pyszora et al, 2017 | I: Physiotherapy programme with myofascial release (MFR) and proprioceptive neuromuscular facilitation (PNF) x 2-3, 30-minute session weekly  C: Usual care  Active exercises, myofascial release, and proprioceptive neuromuscular facilitation (PNF) techniques. | I: *n* = 30  C: *n* = 30  I: mean age= 72.4 yrs  C: mean age= 69.3 yrs Female= 39 (65%) | Mixed Advanced | RCT (1:1) | Hospital | 2 wks | In person  Individual | Primary – BFI, ESAS  Secondary – SS | The physiotherapy program significantly reduced fatigue severity in patients (p<0.01). |
| Quist et al, 2015 | Multidimensional Exercise Intervention x 2, 1.5-hour session weekly  Supervised group training (aerobic, relaxation, strength) in groups of 10 to 12 patients. | *n* = 114  Median age= 66 yrs Female= 57 (50%) | Lung cancer  Stage IIIb-IV | Single-arm interventional study (prospective) | Outpatient clinic | 6 wks | In person  Individual | Primary – Maximal oxygen uptake (VO2 peak)  Secondary – 1-RM, 6MWT | There was a significant increase in aerobic capacity, VO2peak (P < .000) and functional capacity. |
| Quist et al, 2020 | I: Physical exercise intervention (INT)- supervised, structured exercise training x twice weekly. Training included warm-up exercises, strength training, aerobic training, and stretching.  C: Usual care | I*: n*= 110  C: *n* = 108  I: mean age: 72.4 yrs, C: mean age: 69.3 yrs Female: 65 (5.7%) | Lung cancer  Stage IIIb/IV | RCT (prospective) | University hospital | 12 wks | In person  Individual | Primary – Maximal oxygen uptake (VO2 peak)  Secondary – 1-RM, 6MWT | There was no significant difference between the intervention and control group in aerobic capacity VO2 peak. |
| Reljic et al, 2022 | I: Very Low-Volume Interval Training (LOW-HIIT) x 2, 14-minute sessions weekly  C: SHAM group 2 weekly sessions of a 20-minute physical mobilisation. | I: *n* = 13  C: *n* = 14  I: mean age= 53.5 yrs  C: mean age= 58 yrs Female= 14 (51%) | Mixed Advanced | RCT (blinded) | Hospital | 12 wks | In person  Individual | Primary – Attrition and attendance  Secondary – Adverse events | Three patients dropped out during the intervention period (I: n =1, 8% and C: n =2, 14%). |
| Rief & Omlor, 2014 | I: Isometric spinal resistance training x 30 minutes, 5 days for 2 wks, followed by home exercises for another 12 wks  C: did 15-minute respiration exercises and “hot roll” treatments.  Intervention had three different exercises to exercises at home for a further twelve wks. | I: *n* = 30  C: *n* = 30  I: mean age= 61.3 yrs  C: mean age= 64.1 yrs Female= 27 (45%) | Mixed  Advanced bone metastases | RCT (pilot study) | Outpatient clinic | 2 wks + 12 wks FU | In person  Individual | Primary – Completion  Secondary – CT metastases changes | Patients in the intervention group completed the isometric resistance training of the autochthonous muscles in 83.3% (n = 25) of all cases. |
| Rief & Akbar, 2014 | !: Isometric resistance training x 30 minutes, 5 days for 2 wks, followed by home exercise 3 session/week for 6 months. Three exercises to exercises at home for a further twelve wks.  C: 15-minute physical therapy in the form of respiration exercises and “hot roll” treatments. | I: *n* = 30  C: *n* = 30  I: mean age= 61.3 yrs  C: mean age= 64.1 yrs Female= 27 (45%) | Mixed  Advanced bone metastases | RCT (1:1) | Outpatient clinic | 2 wks + 24 wks FU | In person  Individual | Primary – EORTC QLQ  Secondary – EORTC QLQ FA13 | Patients in the intervention group showed a significant improvement in in psychosocial aspects of the EORTC? after three (p = 0.001) and six months (p = 0.010). |
| Rutkowska et al, 2019 | Exercise training program x 5, 30 minutes sessions weekly  Sessions consisted respiratory exercises; specific respiratory exercises; cycle ergometer or treadmill, resistance exercise intensity, Nordic walking, relaxation training. | I: *n* = 26  C: *n* = 14  I: mean age= 58 yrs  C: mean age= 61 yrs Female= 3 (1%) | NSC Lung cancer  Advanced | RCT (2:1) | Hospital | 4 wks | In person  Individual | Primary – 6MWT distance  Secondary – FEV1, FVC | The 6MWT distance improved significantly in the intervention group (p=0.02). |
| Schmidt et al, 2015 | I1: Endurance (ET) x 2, 60-minute sessions weekly  I2: resistance training (RT) x 2, 60-minute sessions weekly  C: Usual care  Strength training tested lower extremities and upper extremities on a chair. The endurance test was assessed as a bicycle ergometer test. | I1: ET: *n* = 20  I2: RT: *n* = 21  C: *n* = 26  I1: mean age= 54 yrs  I2: mean age= 53 yrs  C: mean age= 56 yrs Female= 67 (100%) | Breast Cancer Advanced | RCT (1:1:1 Prospective) | Hospital | 12 wks | In person  Individual | Primary – Isometric muscular capacity with M3 Diagnosis, PWC 250, Borg Scale  Secondary – EORTC QLQ | The muscular strength improved significantly in the RT group for latissimus pull down (p=0.014) and bench press (p=0.012) and ET group bench press (p=0.023). |
| Schuler et al, 2017 | I1: structured individual exercise program taught by the physical therapist.  I2: structured individual exercise program taught by the physical therapist.  And physical therapy treatment twice per week.  C: Exercise program with endurance and strength training x 5, 20–30-minute session weekly. | I1: *n* = 23  I2: *n* = 23  C: *n*= 24  Mean age= 52.38 yrs Female= 29 (41%) | Mixed Advanced | 3- arm RCT | Hospital | 12 wks | In person  Individual | Primary – MFI  Secondary – 6-MWT | There was no significant difference in fatigue for the intervention group. |
| Scott et al, 2018 | I: Aerobic training x 3 sessions a week  C: Individualised stretching x 3 sessions a week  Aerobic training did 36 supervised treadmill walking sessions. Then an individualized stretching session. | I: *n* = 33  C: *n* = 32 Mean age= 54 yrs Female= 100 | Breast Cancer Stage IV | RCT (1:1) | Cancer centre | 12 wks | In person  Individual | Primary – Attendance defined as rate of lost to follow up  Secondary – Adverse events, VO2 peak, SF-36 | 1 of 33 (3%) patients receiving aerobic training was lost to follow up whereas mean attendance was 63%. |
| Stuecher et al, 2019 | I: 3 walking sessions x 20 minutes weekly then 150 minutes a week (inactive participants). 5 walking sessions x 30 minutes weekly to 150 minutes a week (active participants)  C: Usual care | I: *n* = 13  C: *n* = 15  Mean age= 67.1 yrs Female= 19 (43.2%) | GIT Cancer Stage III–IV | RCT (Single blind) | Home | 12 wks | In person  Individual | Primary – SPPB  Secondary – WinFDM capacitive force-measuring platform | There was no significant change in the functional status for the intervention group. |
| Tsianakas et al, 2017 | I: Can Walk intervention 15 min physical motivational telephone interview to motivate 30-minute walking alternate days.  C: Usual care | I: *n* = 21  C: *n* = 21 (control) M: mean age= 65.6 yrs  F: mean age= 59 yrs Female= 21 (50%) | Mixed Advanced | RCT (1:1) | Outpatient clinic | 12 wks | In person  Individual | Primary – ESQ  Secondary – FACT-G | At 24 weeks, nine participants completed the ESQ and results indicated that most (n=8) found it useful and were satisfied (n=7). |
| Uth et al, 2016 | I: Football training x 2, 45-minute session weekly for the first 4 wks; x 2,1-hour session weekly for week 5-8; 3, 1 hour session weekly for week 9-12. Participants played football with 3–7 players a side for on a natural grass pitch or indoors.  C: Normal physical activity | I: *n* = 29  C: *n* = 29  I: mean age= 67.1 yrs  C: mean age= 66.5 yrs Female= 0 | Prostate Cancer Advanced | RCT (1:1) | Football grass pitch | 12 wks | In person  Group | Primary – ESQ  Secondary – FACT-G | A significant difference in total body BMC (26.4 g, 95 % CI: 5.8 to 46.9 g) and leg BMC (13.8 g, 95 % CI: 7.0 to 20.5 g)was observed in the intervention group. |

Notes: I: Intervention; C: Control; 1-RM: hypothetical one-repetition maximum test; 6MWT: 6-min walk test; BDI: Basic dyspnoea index; BFI: Brief Fatigue Inventory; BPI: Brief pain inventory; EORTC-QLQ-C30: European Organization for Research and Treatment of Cancer Quality of Life Questionnaire Core-30; ESAS: Edmonton Symptom Assessment Scale; QoL: quality of life; ESQ: End of study questionnaire; FACIT: Functional Assessment of Chronic Illness Therapy)-Fatigue scale; FACT-B: Functional Assessment of Cancer Therapy – Breast; FACT-G: Functional Assessment of Cancer Therapy-GeneralFEV1: Forced expiratory volume; FVC: Forced vital capacity; HHDM: handheld dynamometry; MFI: Multidimensional Fatigue Inventory; MRC: Modified dyspnoea scale of the Medical Research Council; PWC 150: Physical Worker Capacity Test 150; SF-36: Physical functioning short form; PSI: Pittsburgh Sleep Inventory; SPPB: Short Physical Performance Battery; SS: Satisfaction scores.

**Table 2. Study characteristics of psychosocial interventions (n=20)**

| Author/Year | Intervention | Sample Characteristics | Cancer Type | Study Design | Study Setting | Study Duration | Mode of delivery | Study Outcomes | Key Findings |
| --- | --- | --- | --- | --- | --- | --- | --- | --- | --- |
| Bernatchez et al, 2019 | CBT-E protocol x 1, 60-minute session at home and applied strategies for 3 wks  Individual CBT-E was delivered at home with individualised treatment plan. | *n*= 6 Age range= 57-88 yrs Female= 2 (33%) | Mixed Advanced | Single-arm interventional study | Home | 3 wks | In person  Individual | Primary –TEQ  Secondary – Sleep diary, ISI | TEQ showed that the half of the participants found the intervention very helpful. |
| Breitbart et al, 2015 | I: Meaning-centred group psychotherapy (MCGP) x 8 session: MCGP - develop sense of meaning into their lives.  C: Supportive group psychotherapy (SGP) x one session a week : coping skills by encouraging patients to share concerns, emotions, experiences. | I: *n*= 132  C: *n*= 121  Mean age= 58.2 yrs Female= 186 (69%) | Mixed Advanced | RCT | Cancer centre | 8 wks + 2 month follow up | In person  Group | Primary- SWB  Secondary – BDI, HADS | Significant improvements for intervention (p<0.001) compared with control in spiritual well-being and quality of life. |
| Breitbart W et al, 2018 | I1: Individual meaning-centred psychotherapy (IMCP)  I2: Supportive Psychotherapy (SP), to help patients to share concerns related to their diagnosis and treatment.  C: Provided mental health resources. | I1: *n*= 109  I2: *n*= 108  C: n= 104 Mean age= 58 yrs Female= 230 (71.7%) | Mixed Advanced | 3-arm RCT | Cancer centre | 16 wks | In person  Individual | Primary – SWB  Secondary – HAI, SAHD, HADS-A, HADS-D | Significant improvement in SWB for the intervention (p=0.02). |
| Dose et al, 2018 | Dignity therapy (DT) X 3 interviews and 1 Life plan (LP) session  Nurse led intervention where patients relate important life events to reflect and create a life plan. | *N*= 20 Pancreas: mean age= 63.2 yrs  Lung: mean age= 64 yrs Female= 10 (50%) | Pancreatic or Lung cancer Advanced | Single-arm interventional study (phase I/II pilot) | Outpatient chemotherapy suite | 3 months | In person  Individual | Primary – DT,  Secondary –  FACT-Hep | Distress levels at 3 months post-intervention were significantly lower (p=0.04). |
| Greer JA et al, 2019 | I: Cognitive behavioural therapy (CBT) mobile app x 7 sessions approx. 45 minutes each.  Patient-centred, interactive skills for managing cancer-related anxiety.  C: Health education program. | I: *n*= 72  C: *n*= 73  Mean age= 56.45 yrs Female= 107 (73.8%) | Mixed Stage IV | RCT (1:1) | Home | 12 wks | Online  Individual | Primary – HAM-A  Secondary – HADS, PHQ-9 | The CBT intervention group experienced small changes for anxiety (d= 0.24-0.39). |
| Heidary et al, 2023 | I: Logo therapy x 10, 2-hour sessions for 10 wks. Psychologist led to acquire a greater sense of meaning in their lives by enhancing their motivation for change.  C: Waitlist group were asked not to participate in any non-obligatory spiritual and religious ceremonies. | I: *n*= 31  C: *n*= 32  Mean age= 52.56 yrs Female= 32 (50%) | Mixed Stage III and IV | RCT (blinded) | Outpatient | 10 wks | In person  Individual | Primary – DAS  Secondary – ELQ | Significant effects on lowering death anxiety with logotherapy (p<0.001) but not in control. |
| Li et al, 2022 | I: Acceptance and Commitment therapy x 4 sessions a week (60–90 min/session) and three videoconferencing-based sessions via Tencent video conference.  C: Usual care | I: *n*= 20  C: *n*= 20  Mean age= 56.9 yrs Female= 11 (27.5%) | Lung cancer Stage II/IV | RCT (pilot) | University hospital | 4 wks | Online  Individual | Primary – FSI, FACT-L  Secondary – MFI, PHQ-9, GAD-7 | No changes in fatigue interference but significant changes in health related QoL (p=0.001) |
| Liu et al, 2021 | I: Cognitive behavioural therapy (CBT) + chemoradiotherapy x 6 sessions  C: Usual care | I: *n*= 136  C: *n*= 133  Mean age= 47 yrs Female= 78 (28%) | Nasopharyngeal cancer Stage II/III | Retrospective control trial | Hospital | 6 wks | In person  Individual | Primary – HADS  Secondary – Response rates | Patients in the CBT group showed significantly lower HADS scores (p<0.001). |
| Lloyd-Williams M et al, 2018 | I: DISCERN trial – narrative therapy 25-60 minutes at baseline and followed up at 2.4.6 wks. Patients to discussed sense of meaning regarding depression and their physical, psychological and spiritual well-being.  C: Usual care | I: *n*= 33  C: *n*= 24  Mean age= 65.1 yrs Female= 40 (71%) | Mixed Advanced | RCT (pilot) | Hospice or patient's home | 1 week + 2,4,6 wks follow up | In person  Individual | Primary – PHQ-9  Secondary – ESAS | Intervention had non-significant reduction in PHQ-9 scores. |
| Lo et al, 2014 | Managing Cancer and Living meaningfully (CALM) x 3-8, 60 minutes sessions  Individual session helping with symptom management, communication, relationships, spiritual well-being, preparation for future. | *n*= 50 Mean age= 52.38 yrs Female= 31 (71%) | Mixed Stage III IV | Single arm (pilot) | Cancer centre | 6 months | In person  Individual | Primary – PHQ-9  Secondary – DADDS, SWB | Significant reductions in depressive symptoms (p=0.02) and death anxiety (p=0.02). |
| Lynch FA et al, 2020 | I1: Self-management booklet with cognitive behavioural skills to manage fear of cancer  I2: Individual sessions delivered by a clinical psychologist x 5 sessions (60-90 minutes)  C: Usual care | I1: n= 21  I2: n=7  C: n=22  Mean age= 61.4 yrs Female= 16 (33%) | Melanoma Advanced | RCT | Cancer centre | 16 wks | In person  Individual | Primary – FCR-SF  Secondary – Survivor experience survey | 62% participants (13/21) from the self-management group had a reduction in FCR. At the completion of individual therapy, 5/7 participants had a reduction in their FCR. |
| Mosher et al, 2018 | I: Telephone based Acceptance and Commitment therapy (ACT) x 6 sessions weekly for 50-60 mins.  C: Telephone education/support x 6 sessions  ACT - taught model of behaviour change, including mindfulness, perspective taking, cognitive delusion, acceptance, values clarification, and committed action. | I: *n*= 23  C: *n*= 24  Mean age= 56.25 yrs Female= 100 | Breast cancer Stage IV | RCT (pilot) | University hospital | 6 wks | Online  Individual | Primary – MDASI  Secondary – PROMIS | There was no significant change in indicators of patient symptom interference for ACT and control group. |
| Mosher et al, 2019 | I: Telephone based acceptance and Commitment therapy x 6 sessions weekly for 50 mins.  C: Telephone education/support x 6 sessions  ACT - taught model of behaviour change, including mindfulness, perspective taking, cognitive delusion, acceptance, values clarification, and committed action. | Patient  I: *n*= 25  C: *n*= 25  Mean age= 62.6yrs  Caregiver  I: *n*= 25  C: *n*= 25  Mean age= 57 yrs  Female= 62 (62%) | Lung cancer Stage III/IV | RCT (pilot) | University hospital | 6 wks | Online  Individual | Primary - MDASI  Secondary – PROMIS | No significant changes of patient symptom interference for both patients and caregivers. |
| Murphy MJ et al, 2021 | Cognitive behavioural therapy (CBT) x 6 online lessons over 12 wks  ICBT included four components (lesson, lesson summary, additional information, and the audio-visual component) | *n*= 27 Mean age= 56 yrs Female= 26 (96%) | Mixed Advanced | Single arm open trial | Remote | 12 wks | Online  Individual | Primary – Adherence  Secondary – HADS | High adherence (70% did 4 lessons) and higher for stable patients (85% did 4 lessons). |
| Reb AM et al, 2020 | Conquer Fear videoconferencing sessions with skills practice x 7 sessions via zoom and 1 session in person.  Practiced values clarification exercises, skills practice, self-assessment questionnaires, attention training, planning to manage fears and worries. | *n*= 27  Mean age= 58.9 yrs Female= 26 (83.9%) | Gynaecologic and lung cancer Stage III /IV | Single-arm mixed methods study | Home | 8 wks | Online  Individual | Primary – FOP-SF  Secondary – Impact of event scale-R, PROMIS-anxiety 8a, MCQ-30 | Non-significant improvement of FOP outcome. |
| Rodin G et al, 2018 | I: Managing Cancer and Living Meaningfully (CALM) x 3-6, 45–60-minute sessions over 3-6 months . Therapeutic relationship and reflective space: symptom management and communication with health care providers, changes in self and relations, spiritual well-being, and mortality and future-oriented concerns  C: Usual care | I: *n*= 151  C: *n*= 154 Mean age= 59 yrs Female= 120 (39%) | Mixed Advanced | RCT | Cancer centre | 6 months | In person  Individual | Primary – PHQ-9  Secondary – SCID, FAD-7, DADDS, FACIT-Sp, QUAL-EC | CALM group had significantly lower depressive symptoms at 3 months (p=0.04) and 6 months. (p=0.02) compared to the control. |
| Serfaty et al, 2019 | I: Acceptance and Commitment therapy x 8 weekly 1-hour sessions. ACT - understanding psychopathically elements and learning acceptance.  C: Talk therapy | I: *n*= 20  C: *n*= 22  Mean age= 62 yrs Female= 31 (74%) | Mixed Advanced | RCT | Hospice | 3 months | In person  Individual | Primary - FACT-G  Secondary – K10, AAQII | Adjusted mean difference in total FACT‐G scores was 2.25 (95% CI, −6.03‐10.52) in favour of ACT. |
| Wells-Di Gregorio SM et al, 2015 | I: Cognitive behavioural ‐ acceptance and commitment therapy (CBT‐ACT) x 3, 1.5-hour sessions over 6 wks; sleep diary; interview at the end of the intervention.  C: Waitlist group | I: *n*= 17  C: *n*= 11  Mean age= 56.64 yrs Female= 23 (82%) | Mixed Advanced | RCT (pilot) | Outpatient clinic | 6 wks | In person  Individual | Primary – ISI, PSWQ, IUS, STAI, CES-D, FSI  Secondary – IES-R, JSCS | Significant improvements in sleep efficiency (p=0.0062), sleep latency (p=0.028), worry (0.026), and depression (p=0.03) for the intervention group. |
| Xiao et al, 2013 | I: Nurse-led life review program x 1 session/week for 3 wks. Reviewing the present life, adulthood, and childhood. Then had a life review interview.  C: Usual care | I: *n*= 40  C: *n*= 40  I: mean age= 59.78 yrs  C: mean age= 58.53 yrs Female= 38 (47%) | Mixed Advanced | RCT (Prospective) | Home | 3 wks | In person  Individual | Primary – Single item scale (0 to 10) for QoL  Secondary – QoL Concerns Questionnaire | Significant improvements in the quality of life for the intervention group (p = .000). |
| Yanez B et al, 2015 | I: Cognitive Behavioural Stress Management (CBSM) x 90-minute sessions weekly for 10 wks. Stress reduction and stress management on a website on a tablet.  C: Health promotion. | I: *n*= 37  C: *n*= 37  Mean age= 68.84 yrs Female= 0 | Prostate cancer Stages III-IV | RCT | Outpatient | 10 wks | Online  Individual | Primary – IES-R  Secondary – PROMIS, FACT-G | No significant effects on distress measured with IES-R between groups (p=0.06). |

Notes: I: Intervention; C: Control; AAQII: Acceptance and Action Questionnaire II; BDI: Beck Depression Inventory; CES-D: Centre for Epidemiological Studies Depression Scale (CES‐D); DASS: Depression Anxiety Stress Scale; DADDS: Death and Dying Distress Scale (DADDS); DT: Distress thermometer; ESAS: Edmonton Symptom Assessment; ELQ: Existential loneliness questionnaire; FACT-Hep: Functional Assessment of Cancer Therapy Hepatobiliary; FSI: Fatigue Interference subscale; FCRI-SF: Fear of Cancer Recurrence Inventory Short Form; FACT-L: Functional Assessment of Cancer Therapy–Lung, Version 4; GAD-7: Generalized Anxiety Disorder Scale; HAMA-A: Hamilton Anxiety Rating Scale; HAI: Hopelessness Assessment in Illness questionnaire; HADS-A: Hospital Anxiety and Depression Scale–Anxiety; HADS-D: Hospital Anxiety and Depression Scale–Depression; ISI: Insomnia Severity Index; IUS: Intolerance of Uncertainty Scale; K10: Kessler Psychological Distress Scale; MDASI: MD Anderson Symptom Inventory; MCQ-30: Metacognitions questionnaire; MFI: Multidimensional Fatigue inventory; PHQ-9: Patient Health Questionnaire-9; PSWQ: Penn State Worry Questionnaire; PROMIS: Patient Reported Outcomes Measurement Information System; QUAL-EC: Quality of Life at the End of Life Cancer Scale; SAHD: Schedule of Attitudes Toward Hastened Death; STAI: State‐Trait Anxiety Inventory; SCID: Structured Clinical Interview for DSM-IV-TR Axis I Disorders; JSCS: James Supportive Care Screening; IES‐R: Impact of Event Scale‐Revised.

**Table 3. Study Characteristics for mindfulness-based interventions (n=12)**

| Author/Year | Intervention content | Sample Characteristics | Cancer Type | Study Design | Study Setting | Study Duration | Mode of delivery | Study Outcomes | Key Findings |
| --- | --- | --- | --- | --- | --- | --- | --- | --- | --- |
| Ando et al, 2016 | Mindfulness Art Therapy Short Version (MBAT) x two 1-hour individual sessions  Patients instructed on mindfulness with a CD to assist learning and then made art to express their feelings. | *n*= 10 Mean age= 56 yrs Female= 10 (100%) | Mixed Stage IV | Single-arm interventional study (pilot) | Hospital | 8 wks | In person  Individual | Primary – POMS  Secondary – FACIT-Sp | Total mood score decreased from 28.8 to 17.5. No change for FACIT-Sp. |
| Arden-close et al, 2020 | Mindfulness-based program x 6 group sessions, 1.5 hours and 3, 1 hour focus groups weekly  Meditative and focus group for knowledge, explore experience and views of acceptability. | *n*= 28 Mean age= 59 yrs Female= 28 (100%) | Ovarian cancer  Recurrence at any stage | Single-arm interventional study + focus group | Hospital | 6 wks | In person  Group | Primary – HADS,  Secondary – WEMWBS, FMI, EORTC-QLQ-OV28 | Participants with no reported HADS score ranged from 6% at baseline to 33% at week 12. |
| Chambers et al, 2017 | I: Mindfulness-Based Cognitive Therapy (MBCT) x 1.25 hours followed by 15 minutes meditation weekly. Meditations via phone.  C: Usual care with patient education. | I: *n*= 94  C: *n*= 95  Mean age= 71 yrs Female= 0 | Prostate cancer Advanced | RCT | Home | 8 wks | Online/in person  Individual | Primary – BSI, Impact of event scale, PSA anxiety subscale.  Secondary – FACT-P, PTGI | No significant changes in psychological distress (P= 0.117 and cancer-specific distress (P =0.504) prostate-specific antigen anxiety (P = 0.220). |
| Cheung et al, 2017 | I1: In-person LILAC (lessons in linking affect and coping) 1-hour weekly session. Learned skills to increase the frequency of positive emotions to practice at home.  I2: Online LILAC  C : Interview weekly without didactic portion or skill practice | I1: n= 14  I2: n=12  C: n= 13 Mean age= 53.5 yrs Female= 39 (100%) | Breast cancer Stage IV | 3-arm RCT (blinded) | University | 5 wks | Online/in person)  Individual | Primary – CES- D  Secondary – DES, MQS | LILAC intervention (both in‐person and online) showed statistically significant reductions in depression at the 1 month follow‐up assessment (p = 0.03). Control participants' depression levels remained elevated (p > 0.83). |
| De Paolis et al, 2019 | I: Interactive guided imagery IGI - visualisation of positive and pleasant images and PMR - prolonged deep breathing and relaxation of main muscle groups.  C: Usual care | I: *n*= 53  C: *n*= 51  Mean age= 71.8 yrs  Female= 54 (51.92%) | Mixed Advanced / Metastatic | RCT | Hospice | 4 days | In person  Individual | Primary – NRS  Secondary – ESAS, TSDS | NRS pain score fell from 4.11 at T1 to 2.28 in group A and from 4.51 to 3.96 in group B. |
| Eyles et al, 2015 | Quantitative: Mindfulness-based stress reduction (MBSR) x 1 x week, 120 mins. Body scan, sitting and lying meditation, gentle yoga stretching, mindful walking, and loving-kindness meditation. Focus group to explore acceptability and feasibility.  Qualitative: Face-to-face interviews (once 2 wks before and after 4 months).  Focus group 1-2 hours for nurses (1 month after intervention) | *n*= 20  Age range= 37-65 yrs  Female= 20 (100%) | Breast cancer Advanced | Mixed Methods Study (Non-randomised Intervention + Focus group) | Outpatient clinic | 8 wks | In person  Individual | Primary – BFI  Secondary – HADS, EORTC QLQ C30, TMS | Non-significant reduction in fatigue (p=0.02). |
| Han et al, 2021 | I: Combined Naikan therapy (NT) and Morita therapy (MT) program, NT – 2 hours x 20 sessions. NT examine their relationships. MT had 3 periods: Relative bed rest period (1 week); Light work period (2 wks); Social adaptation training period (1 week).  C: Usual care | I: n= 65  C: n= 65  Mean age= 58 yrs Female= 86 (66%) | Mixed Advanced | RCT (1:1 assessor blinded) | Hospital | 7 wks | In person  Individual | Primary – DT  Secondary – DT problem list | Intervention decreased psychological aspects of the DT significantly (p<0.001). |
| Lee et al, 2017 | Mindfulness-based stress reduction (MBSR) x 120 mins weekly. Body scan, meditation, group discussions, and mindfulness in communication and everyday life.  C: Waitlisted group | I: *n*= 9  C: *n*= 9  I: mean age= 52 yrs,  C: mean age= 57 yrs Female= 18 (100%) | Breast cancer Advanced | RCT | Outpatient clinic | 8 wks | In person  Individual | Primary – BPI  Secondary – HRV | Non-significant decrease in average pain (p=0.08). |
| Milbury et al, 2020 | I1: Couple-based meditation (CBM) CBM on interconnection, mindfulness, and compassion.  12: supportive-expressive (SE) -– 60 minutes weekly via Facetime. SE on discussing and sharing cancer-related concerns and share their concerns.  C: Usual care | I1: P: *n*= 26  I2: P: *n* = 24  C: P: *n* = 25  I1: S: *n*= 26  12: S: *n*= 24  C: S: *n*= 25  P: Mean age= 65 yrs  S: Mean age = 63.9 yrs  Female= 77 (51%) | Non-small cell lung cancer (NSCLC)  Stage IV | RCT | Online | 4 wks | In person  Group | Primary – CES-D,  Secondary – IES, SWB | No significant improvements in depression (p-0.95). |
| Mosher et al, 2018 | I1: Peer helping + coping skills intervention (telephone-based) x 5, 50–60 minutes, help create an informational resource on quality-of-life issues  I2: Coping skills, discussed the same topic but did not help create a resource | Patients  I1: *n*= 25  I2: *n*= 25  Caregivers  I1: *n*= 25  I2: *n*= 25  Mean age= 58.88 yrs Female= 52 (52%) | GIT cancer Stage IV | RCT | Cancer centre | 2 wks | In person  Individual | Primary – FACIT-Sp  Secondary – PROMIS  . | PH + coping skills group did not experience higher levels of meaning in life and peace than the coping skills group. It was stable over time for the PH + coping skills group and showed a small increase in the coping skills group. |
| Poletti et al, 2019 | Mindfulness-based stress reduction (MBSR) x 8, 2.5-hour session/week, 4.5-hour session from week 6-7-, and 30-minute home practice daily  Formal sitting meditation, body scan, light yoga, walking meditation, and Aikido exercises. | *n*= 20  Mean age= 54 yrs  Female= 17 (85%) | Mixed Advanced / metastatic | Mixed-methods study (single arm, + focus group) | Hospital | 8 wks | In person  Individual | Primary – NRS  Secondary – POMS | Non-significant decrease in pain scores (p=0.76) but significant decrease in POMS (p=0.01). |
| Zimmermann et al, 2020 | Coping with Cancer Mindfully intervention x 4, 20-minute sessions weekly for 4 wks  One on one guided meditation (with breathing and relaxation techniques). | *n*= 20  Mean age= 55.6 yrs  Female= 15 (75%) | Mixed Stage III or IV | Single-arm interventional study | Outpatient | 4 wks | In person  Individual | Primary – MCS  Secondary – AAQ-II, MLQ | Mindfulness coping scores increased significantly (p<0.01). |

Notes: I: Intervention; C: Control; P: Patient; S: Spouse; AAQ-II: Acceptance and Action Questionnaire-II; BFI: Brief fatigue inventory; BPI: Brief pain inventory**;** BSI: Brief symptom inventory**;** CES-D: Centre for epidemiological studies depression scale; DES: Differential Emotions Scale**;** DT: Distress Thermometer; EORTC QLQC30: European Organisation for Research and Treatment of Cancer Quality of Life Questionnaire;

EORTC-QLQ-OV28: European Organisation for research and treatment of cancer-quality of life questionnaire – Ovarian cancer module; ESAS: Edmonton Symptom Assessment Scale; FACIT-Sp: Functional assessment of chronic illness therapy- spiritual well-being 12; FACT- P: Functional Assessment of Cancer Therapy-Prostate; FMI: Freiburger mindfulness inventory; HADS: Hospital anxiety and depression scale; HRV: Heart rate variability; IES: Impact of events scale; MCS: Mindful Coping Scale; MLQ: Multifactor leadership questionnaire; MQS: Multidimensional Quality of Life Scale—Cancer Version; NRS: Numeric rating scale; POMS: Profile of Mood States; PROMIS: Patient-reported outcomes measurement information; PTGI: Posttraumatic Growth Inventory; SWB: Spiritual well-being scale; TMS: Toronto Mindfulness Scale; TSDS: Total Symptom Distress; WEMWBSS: Warwick-Edinburgh mental well-being scale.

**Table 4. Patient care and autonomy (n=8)**

| Author/Year | Intervention | Sample Characteristics | Cancer Type | Study Design | Study Setting | Study Duration | Mode of delivery | Study Outcomes | Key Findings |
| --- | --- | --- | --- | --- | --- | --- | --- | --- | --- |
| Crafoord et al, 2020 | Interaktor app instructed patients to report their symptoms daily. Telephone interviews were conducted with patients after.  I1: Breast Cancer  I2: Porstate Cancer | I1: *n*= 74  I2: *n*= 75  I1: median age= 47 yrs  I2: median age= 75 yrs Female= 74 (50%) | Prostate or Breast cancer Advanced | Mixed Methods study | University hospitals, Home | 18 wks breast, 9 wks prostate | Online  Individual | Primary – Engagement  Secondary – Adherence | Engagement the breast cancer group ranged from 22 to 183 days. The prostate cancer group ranged from 54 to 89 days (groups had different treatment duration). |
| Kim H and Kim S et al, 2018 | I: Mobile game group played the education game for >30 minutes a day 3 times a week.  C: read educational material >30 minutes 3 times a day. | I: *n*= 36  C: *n*= 40  I: Median age= 49.8 yrs  C: Median age= 52.1 yrs Female= 100 | Breast cancer Advanced / Metastatic | RCT | Home | 3 wks | Online  Individual | Primary – Time spent game playing  Secondary –K-MARS | Time spent on game in the intervention was higher than that spent for self-education in the control (P<.001). |
| Kim H et al, 2013 | I: Telemonitoring regarding pain x daily for 1 week by Nurse practitioner  C: Pain education | I: *n*= 54  C: *n*= 54  Median age= 59.8 yrs Female= 35 (32%) | Mixed Stage IV | RCT (blinded) | Outpatient clinic | 1 week | Online  Individual | Primary – BPI  Secondary – HADS, DT, EORTC QLQ-C30 | Intervention improved pain scales at 1 week, including worst (p <0.01) and average pain (p <0.01). |
| Lai-Kwon et al, 2022 | I: Nurse-led tele-health delivered survivor ship care (MELCARE) 2 x 1-hour consultations 3 months apart | I: *n*= 30  Median age= 67 yrs Female= 10 (32%) | Melanoma Stage II or Stage IV | Mixed methods study | Melanoma Institute | 3 months | Online  Individual | Primary – DT  Secondary – Rates of consent and study completion | DT was reduced from 5.6 to follow-up 1.5. 31/54 people consented. 97% completion. |
| Petzel et al, 2018 | I: Patient-centred information-based website "Together" x 2-3 times a week that used learning and practicing stress and distress management skills.  C: Usual care | I: *n*= 20  C: *n*= 15  Mean age= 57.3 yrs Female= 100 | Ovarian cancer Advanced | RCT | Home | 60 days | Online  Individual | Primary – Knowledge using a 10-item true-false scale  Secondary – HADS | No significant difference in knowledge score and distress measures. |
| Schuurhuizen et al, 2019 | I: Targeted selection, enhanced care, stepped care program (TES). Watchful waiting, guided self-help program via Internet, booklet, face-to-face problem-solving offered by a trained nurse, and referral to psychosocial services or psychotropic medication.  C: Usual care | I: *n*= 184  C: *n*= 165  Mean age= 66.1 yrs Female= 125 (35.8%) | Colorectal cancer  Advanced | RCT (cluster) | Hospitals | 18 wks | In person Individual | Primary – HADS  Secondary – EORTC QLQ-C30 | No significant changes for the intervention and control group in anxiety, depression, and distress. |
| Voruganti et al, 2017 | I: Loop: a secure online communication tool for team-based clinical collaboration  C: Usual care | I: *n*= 24  C: *n*= 24  I: mean age= 60 yrs C: mean age= 59.5 yrs Female= 8 (69%) | Mixed Stage III or IV | Nonblinded, pragmatic pilot cluster-RCT | Hospital | 3 months | Online Individual | Primary – Recruitment  Secondary – PCCS, POS, ESAS | Mean number of patients recruited per physician was similar (intervention 2.4 [range 0-7], control arm 2.7 [range 0-7]). |
| Ye et al, 2017 | I: Be Resilient to Breast Cancer’ (BRBC) program x 120 min/ week for 12 months. Education program and group discussion, led by clinical psychologists, dietician, Chinese medicine practitioner, to help patients gain a sense of control in their life.  C: CD relaxation therapy and monthly telephone follow-up | I: *n*= 95  C: *n*= 85  I: mean age= 26 ≤ 40 yrs old, 47 > 40 and ≤ 60, 22> 60;  C: mean age= 24 ≤ 40 yrs old, 35 > 40 and ≤ 60, 26> 60 Female= 100 | Breast cancer Advanced/ metastatic | RCT (1:1) | Hospital | 12 months | In person Individual | Primary – 3- and 5-year survival  Secondary – HADS, EORTC QLQ-C30, ALI | The median 5-year survival was 36.7 months (95% CI, 32.9–40.5) in the intervention group and 31.5 months (95% CI, 28.8–35.9) in the control group. The Log Rank (Mantel-Cox) for 3-year survival (p= 0.183) and 5-year survival (p= 0.075) were not significant. |

Notes: B: Breast; P: Prostate; I: Intervention; C: Control; BPI: Brief pain inventory; ALI: Allostatic Load Index; DT: Distress Thermometer; EORTC QLQC30: European Organisation for Research and Treatment of Cancer Quality of Life Questionnaire; ESAS: Edmonton Symptom Assessment Scale; HADS: Hospital Anxiety and Depression Scale; K-MARS: Korean version of the Medication Adherence Rating Scale; PCCS: Picker Continuity and Coordination subscale questionnaire; POS: Palliative care Outcomes Scale.

**Table 5. Study characteristics for electrical-stimulation and acupuncture interventions (n=6)**

| Author/Year | Intervention content | Sample Characteristics | Cancer Type | Study Design | Study Setting | Study Duration | Mode of delivery | Study Outcomes | Key Findings |
| --- | --- | --- | --- | --- | --- | --- | --- | --- | --- |
| Cheung et al, 2022 | I: Self-administered acupressure x 7-hour training week 1 to 4 + self-practice 2 x15 min a day. Two 2-hour training sessions in the first week and self-administered 4 different acupoints.  C: heath talk. | I: *n*= 15  C: *n*= 15  Mean age I= 61.8yrs, Mean age C= 59.93yrs Female= 24 (80%) | Mixed  Stage III/IV | RCT (1:1 pilot) | Home or University hospital | 4 wks | In person Individual | Primary – BFI  Secondary – BPI, HADS, FACT-G | No significant changes in cancer related fatigue. |
| Guo and Wang, 2018 | I: Nerve electrical stimulation (NES) x everyday 30 mins NES at bilateral acupoints.  C: sham - gel pads on without stimulation. | I: *n*= 62  C: *n*= 62  I: mean age= 62.1yrs,  C: mean age= 60.7yrs  Female= 53 (42%) | Gastric cancer Advanced | RCT (1:1 blinded) | Hospital | 1 wk | In person Individual | Primary – VAS  Secondary – MDASI | Significant reduction of nausea (P = 0.02) and vomiting (P =0 .04), and improvement of appetite (P = 0.02). |
| Jeon et al, 2017 | I: True Moxibustion acupuncture x 5 times weekly  C: sham moxibustion x 5 times weekly  Same 5 acupoints (CV12, CV8, CV4, and ST36). | I: *n*= 9  C: *n*= 7 Mean age= 60.3yrs Female= 3 (16%) | Mixed Advanced | RCT (1:1) | Outpatient clinic | 2 wks | In person Individual | QoL – FAACT  Secondary – EORTC QLQ-C30, VAS | Intervention scored lower on the FAACT (14.67 ± 9.73) vs control (26.57 ± 9.50). |
| Kim and Lee, 2018 | I: Intradermal Acupuncture (IA) x twice a day. CV12, bilateral ST25, LI4, LR3, PC06, and Ashi points.  C: Sham (blunt needle). | I: *n*=15  C: *n*= 15  Mean age= 56.1yrs  Female= 11 (36%) | Mixed Advanced | RCT (1:1 blinded) | Outpatient clinic | 3 wks | In person Individual | Primary – Change in analgesics dose  Secondary – NRS | Non-significant reduction in analgesic use for the intervention (p=0.180). |
| Nakano et al, 2020 | Transcutaneous electrical nerve stimulation (TENS)- First period x every day 5 days 30 mins wash-out and second period everyday 5 day 30 mins. | *n*= 24  Mean age= 79yrs Female= 3 (15%) | Mixed Advanced | Randomised crossover pilot study | Hospital | 10 days | In person Individual | Primary – NRS  Secondary – EORTC, QLQ-C15-PAL | TENS significantly reduced pain immediately (p<0.01) but no lasting effect after 60 (p=0.26) and 120 min (p=0.99). |
| Zhang et al, 2018 | Neuromuscular Electrical Stimulation (NMES) x 2 times a week 30 mins. At bilateral Zusali (ST36) intensity increased to tolerance  C: Usual care | I: *n*= 30  C: *n*= 30  I: mean age= 64.2yrs  C: mean age= 63.9yrs Female= 21 (36%) | Breast cancer Advanced | Retrospective control trial | Hospital | 8 wks | In person Individual | Primary – MFI  Secondary – HADS, PSQI | No significant reduction in cancer fatigue relief (p = 0.21). |

Notes: I: intervention; C: control; BFI: Brief Fatigue Inventory; BPI: Brief pain inventory; QLQ-C15-PAL: Quality of life questionnaire Core 15 Palliative scores; FAACT: Functional Assessment of Anorexia/Cachexia Therapy; FACT-G: Functional Assessment of Cancer Therapy General; Hospital Anxiety and Depression Scale (HADS); MDASI: MD Anderson Symptom Inventory (MDASI); NRS: Numerical rating scale; PSQI: Pittsburgh Sleep Quality Index; VAS: Visual analogue scale; MFI: Multidimensional Fatigue Index; EORTC QLQ-30: European Organisation for Research and Treatment of Cancer Core Quality of Life questionnaries-30.

**Table 6. Study characteristics of multimodal interventions (n=6)**

| Author/Year | Intervention content | Sample Characteristics | Cancer Type | Study Design | Study Setting | Study Duration | Mode of deiivery | Study Outcome | Key Findings |
| --- | --- | --- | --- | --- | --- | --- | --- | --- | --- |
| Bourke et al, 2014 | I: At least 1 independent exercise session (e.g. cycling, gym) x 30 mins in first 6 wks, increased to twice a week during wk 7-12. Group seminar for exercise goals and nutrition x 20 mins every 2 wk  C: Usual care | I: *n*= 50  C: *n*= 50  Mean age= 71 yrs Female= 0 | Prostate cancer Advanced | 2-arm single-blind RCT design (1:1) | Outpatient clinic | 12 wks | In person Individual/Group | Primary - FACT-P, Diastolic blood pressure change  Secondary – FACT-F | Significant improvements in FACT-P at the end of the supervised intervention (adjusted mean difference: 8.9 points; 95% CI, 3.7–14.2) but not sustained after (adjusted mean difference: 3.3 points; 95% CI, -2.6 to 9.3) |
| Kim et al, 2016 | Wheel Balance Cancer Therapy (WBCT) consisting of diet, acupuncture, yoga, and mediation daily.  Dietary advice: green vegetables and fruit juice 2x/day; acupuncture, moxibustion, hydrotherapy, and herbal hot pack therapy. Meditation, controlled breathing, yoga. | *n*= 33 Mean age= 63.2 yrs Female= 10 (30.3%) | Lung cancer Advanced | Single-arm interventional study | Inpatient cancer centre | 2 wks of intervention  F/Up: 13 years | In person Individual | Primary – OS  Secondary – Adverse event | OS rates were 63.6% and 24.2% at the ends of years 1 and 2, respectively. |
| Lacey et al, 2018 | I: Immunotherapy with supportive care intervention with 16 exercise, dietary advice, non-invasive complementary therapies, psychology consultation.  C: Immunotherapy / usual care | I: *n*= 13  C: *n*= 15  Mean age= 66 yrs Female= 12 (43%) | Metastatic melanoma | RCT | Cancer centre | 12 weeks | In person Individual | Primary – Adherence  Secondary –ESAS | Overall adherence was 85%. Symptoms most troubling at baseline were fatigue, sleep, general aches, memory. There were no significant changes. |
| Poort et al, 2020 | I1: Cognitive behavioural therapy (CBT) x 10, 1-hour sessions. Modules on different fatigue-perpetuating cognitions and behaviours.  I2: Graded exercise therapy (GET) x 2-hour weekly session. Individually graded aerobic and resistance training.  C: Usual care | I1: *n* = 46  I2: *n* = 42  C: *n* = 46  Mean age= 62.76 yrs Female= 77 (57%) | Mixed Advanced / metastatic | 3 arm- RCT | Hospital | 26 wks | In person Individual | Primary – CIS Fatigue  Secondary – EORTC-QLQ-C30 | CBT significantly reduced fatigue (p=0.012) between ysyak care. GET vs usual care was not statistically significant. |
| Schink et al, 2018 | I: Whole-body electro-myo-stimulation (WB-EMS training) x 2, 20 minutes sessions and monitored nutrition. Light dynamic physical exercises. Electrical muscle stimulation 6s on and 4s rest.  C: Usual care Nutritional intake pf daily protein intake of > 1.0 g/kg for both groups. | I: *n*= 96  C: *n*= 35  I: mean age= 60.3 yrs  C: mean age= 59.1 yrs Female= 57 (59%) | Mixed Stage III/IV | RCT (pilot) | Hospital | 12 wks | In person Individual | Primary – Muscle mass  Secondary – Body weight (kg) | WB-EMS had a significantly higher skeletal muscle mass at 12 wks (p=0.022). |
| Zimmer et al, 2017 | I: Exercise program, including endurance, resistance, and balance training x twice a week.  C: Waitlist group  60 mins program- Phase I: Balance training (10 mins), Coordination practice (5 mins); Phase II: Endurance training (10 mins), Resistance training (20 mins); Phase III: Cool down (10-15 mins). | I: *n*= 17  C: *n*= 13  I: mean age= 68.53 yrs  C: mean age= 70 yrs Female= 9 (30%) | Colorectal Cancer Stage IV | RCT | Sports Centre | 8 wks | In person Individual | Primary – TOI  Secondary – GGT-Reha | Significant differences between intervention and control in the TOI from baseline to t1 (p=0.028) and t2 (p=0.031) but not from t1 to t2 (p=0.592). |

Notes: I: Intervention; C: control; CIS-Fatigue: Central Sensitisation Inventory; EORTC-QLQ-C30: European Organisation for Research and Treatment of Cancer Quality of Life Questionnaire; FACT- P: Functional Assessment of Cancer Therapy-Prostate; FACT-F: Functional Assessment of Chronic Illness Therapy – Fatigue; GGT-Reha: Gleichgewichtstest; OS: Overall Survival; TOI: Trial Outcome Index.

**Table 7. Study characteristics of other interventions (n=4)**

| Author/Year | Intervention | Sample Characteristics | Cancer Type | Study Design | Study Setting | Study Duration | Mode of delivery | Study Outcomes | Key Findings |
| --- | --- | --- | --- | --- | --- | --- | --- | --- | --- |
| Foucre et al, 2022 | I: Yarrow liver compress x daily for 2 wks, 7 times minimum.  C: Usual care | I: *n*= 10  C: *n*= 10  Median age= 57 yrs Female= 15 (75%) | Mixed Advanced / Metastatic | RCT (1: 1 prospective pilot) | Hospital | 2 wks | In person Individual | Primary - HRV  Secondary – MFI | There were no significant differences in HRV except for t2d (at the end during daytime). |
| Ghadjar P et al, 2021 | I: Yarrow liver compresses with palliative radiation therapy (RT) x 1 time a day.  a total of 7 times.  C: Palliative radiation therapy | I: *n*= 19  C: *n*= 20  Median age= 58.5 yrs Female= 18 (75%) | Mixed Advanced / metastatic | RCT (prospective pilot) | Hospital | 2 wks | In person Individual | Primary – MFI  Secondary – VAS | Non-significant improvement in fatigue in the intervention group (p = 0.13). |
| Lin et al, 2017 | I: Individualised diets continuously adjusted by a dietitian according to nutritional status.  C: Ordinary diet guidance | I: *n*= 55  C: *n*= 55  I: mean age= 52.61 yrs C: mean age= 54.8 yrs Female= 100 | Colorectal cancer Advanced | RCT (prospective) | Hospital | 1 week | In person Individual | Primary – Weight (kg)  Secondary – Serum albumin and prealbumin | Significant change with intervention in patient weight and serum albumin and prealbumin levels (p<0.05). |
| Warth et al, 2015 | I: Live music–based relaxation exercises x 2, 30 minutes sessions two days apart  C: 20-minute Mindfulness-Based Stress Reduction (MBSR) Program x 20 minutes daily | I: *n*= 42  C: *n*=42  Mean age= 63.0 yrs  Female= 60 (71.4%) | Mixed Advanced | Blinded-RCT | Hospital | 3 days | In person Individual | Primary – VAS  Secondary – HRV | Self-rated relaxation (p<0.001) and well-being scores (p=0.013) showed significantly greater increases in the music therapy group. |

Notes: I: Intervention; C: Control; HRV: Heart rate variability; MFI: Multidimensional fatigue inventory; VAS: Visual analogue scale.

**Table 8. Study characteristics for yoga interventions (n=2)**

| Author/Year | Intervention | Sample Characteristics | Cancer Type | Study Design | Study Setting | Study Duration | Primary outcome & Intervention content | Mode of delivery | Key Findings |
| --- | --- | --- | --- | --- | --- | --- | --- | --- | --- |
| Milbury et al, 2015 | I: Couple based Vivekananda Yoga (VKC) program x 2-3 weekly, 60-minute sessions over 6 wks. Joint loosening with breath synchronization, deep relaxation, breath energization (pranayama) with sound resonance, and meditation.  C: Usual care | I: *n*= 15  C: *n*= 15 Patient mean age= 73 yrs, Caregiver mean age= 62 yrs Female= 10 (63%) | Lung cancer Stage III | Single-arm interventional trial (pilot) | Outpatient clinic | 6 wks | Primary – BSI  Secondary – MOS-SF 36 | In person  Group | For patients there was as significant decrease in anxiety (p=0.04) and non-significant decrease for caregivers. |
| Porter et al, 2019 | I: Mindful yoga x 8, 120-minute weekly group sessions. Gentle postures, breathing techniques, meditation, education, and group discussions. Support group sessions on discussion of issues relevant to patients coping with MBC.  C: Social support group | I: *n*= 40  C: *n*= 20  Mean age= 57.3 yrs Female= 100 | Breast cancer Advanced | RCT (2:1) | Outpatient clinic | 8 wks | Primary – CSQ-8  Secondary – BPI, BFI, PSQI, HADS, FFMQ-SF, 6WMT | In person  Group | 82% (28/34) of participants in the yoga group and 65% (11/17) in the control group reported high intervention satisfaction. |

Notes: I: Intervention; C: control; 6WMT: 6-minute walk test; BFI: Brief Fatigue Inventory; BPI: Brief Pain Inventory-Short Form; BSI: Brief Symptom Inventory; CSQ- 8: Client Satisfaction Questionnaire-8; FFMQ-SF: Five Facet Mindfulness Questionnaire-Short Form; HADS: Hospital anxiety and depression scale; MSO-SF 36: Medical Outcomes Study 36-item Short-Form; PSQI: Pittsburgh Sleep Quality Index.
