# Supplementary material for: Living well with advanced cancer: a scoping review of non-pharmacological supportive care interventions
Source: J Cancer Surviv. 2024 Dec 16;20(3):1119–30. doi: 10.1007/s11764-024-01714-z (PMC13144243; doi:10.1007/s11764-024-01714-z)
Supplement: Supplementary file 3 — Supplementary file3 (DOCX 20 KB) [file 11764_2024_1714_MOESM3_ESM.docx]

**Supplementary Material 2: Search Strategy**

| 1. | Neoplasm Metastasis/ |
| --- | --- |
| 2. | (cancer* or neoplas* or tumo?r* or malignan* or carcinoma* or metasta* or leuk?emi* or lymphoma* or myeloma* or oncolog* or sarcoma*).ti,kw. |
| 3. | (advance* or progress* or incurab* or inoperab* or recur* or palliative*).mp. [mp=title, book title, abstract, original title, name of substance word, subject heading word, floating sub-heading word, keyword heading word, organism supplementary concept word, protocol supplementary concept word, rare disease supplementary concept word, unique identifier, synonyms] |
| 4. | 2 and 3 |
| 5. | 1 or 4 |
| 6. | Counseling/ or Patient Education as Topic/ or Needs Assessment/ or "Health Services Needs and Demand"/ |
| 7. | (service* or program* or intervent* or education or class* or educat* or resource or rehabilit* self-manag* or rehabilit* self eff* or treatment*).tw,kf. |
| 8. | (Supportive adj1 (care or therap*)).mp. [mp=title, book title, abstract, original title, name of substance word, subject heading word, floating sub-heading word, keyword heading word, organism supplementary concept word, protocol supplementary concept word, rare disease supplementary concept word, unique identifier, synonyms] |
| 9. | 6 or 7 or 8 |
| 10. | COST OF ILLNESS/ |
| 11. | ((cost* or financ* or unemploy* or employ* or work*) adj2 (toxicity or distress or stress or strain or hardship or advice or need* or assistance or barrier* or impact or burden* or effect or worry or worries or support or poverty or difficult* or consequences or income) adj2 patient*).tw,kf. |
| 12. | ("out-of-pocket costs" or "out-of-pocket expenses" or co-pay*).tw,kf. |
| 13. | 10 or 11 or 12 |
| 14. | (Sex counseling or sex counselling).tw,kw. |
| 15. | Sex Counseling/ |
| 16. | Sex Education/ |
| 17. | sex educat*.ti,kw. |
| 18. | psychosexual therapy.tw,kw. |
| 19. | 14 or 15 or 16 or 17 or 18 |
| 20. | nutrition therapy/ or exp diet therapy/ or nutritional support/ |
| 21. | ((diet* or Food or Eat* or Nutrit* or Weight or Energy) adj4 (Therapy or therapies or service* or regime* or evaluation or guide*)).tw,kf. |
| 22. | 20 or 21 |
| 23. | exp Exercise/ or exp "Physical Education and Training"/ or Sedentary Behavior/ or exp Sports/ or exp physical therapy modalities/ or exercise movement techniques/ or yoga/ or exp exercise therapy/ or exp musculoskeletal manipulations/ or therapy, soft tissue/ or exp acupressure/ |
| 24. | exp Acupuncture Therapy/ or exp Acupuncture Points/ or exp Acupuncture, Ear/ or acupuncture.mp. or exp Needles/ or needl*.mp. or electroacupuncture.mp. or exp Electroacupuncture/ or acupoint*.mp. or acupressure.mp. or exp Acupressure/ or Zhen Jiu.mp. or Moxibustion.mp. or exp Moxibustion/ or Moxa.mp. or Artemisia/ or Mugwort.mp. or exp Artemisia/ or exp Meridians/ or Meridian*.mp. or Meridian therapy.mp. or Oriental medicine.mp. |
| 25. | ((physical or exercise* or aerobic* or anaerobic*) adj4 (therap* or activit* or inactivit* or train* or educ* or class or regime or program or fit* or movement or techniq* or test*)).tw,kf. |
| 26. | (Sport* or physiotherap* or physio* or kinesiotherap* or massage or yoga or acupressure).tw,kf. |
| 27. | rehabilitation/ or "activities of daily living"/ or occupational therapy/ |
| 28. | 23 or 24 or 25 or 26 or 27 |
| 29. | 22 or 28 |
| 30. | Patient Education as Topic/ or Health Education/ or Consumer Health Information/ or Information Services/ |
| 31. | ((patient or consumer or client) adj3 (Material* or Program* or Session* or Communicat* or Counsel* or Service* or office visit* or Referral* or consult* or telephon* or phon* or voice* or hotline or hot line or messag* or system* or technolog* or computer* or call* or visit* or video* or tape* or audio* or sms or text messag* or Chatroom* or Chat room* or Bulletin board or Social network or Electronic mail or DVD or CD or film* or online or internet or telecommunication* or website* or mail* or email* or blog* or podcast* or portal or computer program* or computer based or computer assisted or web or material* or booklet* or pamphlet* or leaflet* or brochure* or flyer* or poster* or diar* or communic* or guidebook or sheet* or checklist or check list or helpcard)).tw,kf. |
| 32. | 30 or 31 |
| 33. | Patient Care Team/ or Patient Care Planning/ or Patient Care/ or "Continuity of Patient Care"/ or "Delivery of Health Care, Integrated"/ or "Delivery of Health Care"/ or Practice Patterns, Nurses'/ or Patient-Centered Care/ |
| 34. | (continuity of patient care* or care continuum or continuity care or Care continuity or Continuum of care or case manage* or care manage* or nurse lias* or lias* nurse or patient care* plan* or Patient care team or health care management or health care planning or Community Health Planning).tw,kf. |
| 35. | (nurse-led or "nurse led").tw,kf. |
| 36. | (Patient centered care or patient centred care).tw,kf. |
| 37. | ((collaborat* or multidisciplin* or interdisciplin* or team or shared or integrat* or multi agen* or multi-agen*or inter agen* or inter-agen*or Cooperat* or co-operat* or Share* or Interpersonal*) adj2 (Practice* or Care or Team or Service* or treatment* or working or health care or health care system or Continuity or communication)).tw,kf. |
| 38. | 33 or 34 or 35 or 36 or 37 |
| 39. | 32 or 38 |
| 40. | "Religion and Medicine"/ or "religion and psychology"/ or pastoral care/ or spirituality/ |
| 41. | (miracle or faith or god or prayer* or religio* or spiritual*).tw,kf. |
| 42. | 40 or 41 |
| 43. | "Quality of Life"/ |
| 44. | ((Emotion* or Social or Family or Psychological or Cogniti* or Relational or Famil* or Marital or Psychosocial or Psycho-social or Mental health or psychiatric* or relaxation or psycho-analytic or psychoanalytic) adj3 (Wellbeing or Well being or Well-being or Function* or difficult* or dysfunction* or support or therap* or services or technique or training or intervention or Quality of life or quality-of-life or QOL or health related quality of life or health-related quality of life or health related quality-of-life or health-related quality-of-life or HQOL or HRQOL)).tw,kf. |
| 45. | Self-Help Groups/ or Complementary Therapies/ or Mind-Body Therapies/ or Behavior Therapy/ or Counseling/ or exp Psychotherapy/ or Alternative Medicine/ |
| 46. | (group therap* or Cognitive Therapy or Behavio?r Therapy or Counsel* or cognitive behavio* therapy or cognitive behavio* technique or psychotherapeutic or CBT or "acceptance and commitment therapy" or self manag* or self help group* or mind body therap* or complementary therap* or complementary med* or alternative therap* or alternative med* or hypnotherapy or Hydrotherapy or Support group* or Mind body* or Body mind* or Mindfulness based* or social support or social work).tw,kf. |
| 47. | exp tai chi/ or Tai chi.mp. or Tai ji.mp. or exp yoga/ or yog*.mp. or Asana*.mp. or Pranayama.mp. or Dharana.mp. or Dhyana.mp. or exp Mind-body therapies/ or mind-body therapies.mp. or exp Mindfulness/ or mindfulness*.mp. or MB*.mp. or exp Meditation/ or Meditat.mp. or exp relaxation therapy/ |
| 48. | (psychotherap* or psycholog* or hypnotherap* or psychodynamic or psychoeducat* or psycho-educat*).tw,kf. |
| 49. | Interview, psychological/ or exp Adaptation, Psychological/ |
| 50. | (cope or coping).tw,kf. |
| 51. | (aromatherap* or aroma therap* or homeopath* or homeopath* therap* or ayurved*).mp. [mp=title, book title, abstract, original title, name of substance word, subject heading word, floating sub-heading word, keyword heading word, organism supplementary concept word, protocol supplementary concept word, rare disease supplementary concept word, unique identifier, synonyms] |
| 52. | Aromatherapy/ or Homeopathy/ or Medicine, Ayurvedic/ |
| 53. | 43 or 44 or 45 or 46 or 47 or 48 or 49 or 50 or 51 or 52 |
| 54. | 13 or 19 or 29 or 39 or 42 or 53 |
| 55. | 5 and 9 and 54 |
| 56. | limit 55 to (english language and full text and humans and yr="2013 -Current") |
